# Reproduction of East-African bats may guide risk mitigation for coronavirus spillover

**DOI:** 10.1186/s42522-019-0008-8

**Published:** 2020-02-07

**Authors:** Diego Montecino-Latorre, Tracey Goldstein, Kirsten Gilardi, David Wolking, Elizabeth Van Wormer, Rudovick Kazwala, Benard Ssebide, Julius Nziza, Zikankuba Sijali, Michael Cranfield, PREDICT Consortium, Jonna A. K. Mazet

**Affiliations:** 1 One Health Institute, School of Veterinary Medicine, University of California, Davis, CA USA; 2 Gorilla Doctors, Mountain Gorilla Veterinary Project Inc, Davis, CA USA; 3 Institute of Agriculture and Natural Resources, School of Natural Resources, University of Nebraska, Lincoln, NE USA; 4 College of Veterinary Medicine and Biomedical Sciences, Sokoine University of Agriculture, Morogoro, Tanzania; 5 Gorilla Doctors, Mountain Gorilla Veterinary Project Inc., Kampala, Uganda; 6 Gorilla Doctors, Mountain Gorilla Veterinary Project Inc., Musanze, Rwanda; 7 https://ohi.vetmed.ucdavis.edu/programs-projects/predict-project/authorship

**Keywords:** Bats, Coronavirus, Shedding, Seasonal, Reproductive cycle, Weaning, East-Africa

## Abstract

**Background:**

Bats provide important ecosystem services; however, current evidence supports that they host several zoonotic viruses, including species of the *Coronaviridae* family. If bats in close interaction with humans host and shed coronaviruses with zoonotic potential, such as the Severe Acute Respiratory Syndrome virus, spillover may occur. Therefore, strategies aiming to mitigate potential spillover and disease emergence, while supporting the conservation of bats and their important ecological roles are needed. Past research suggests that coronavirus shedding in bats varies seasonally following their reproductive cycle; however, shedding dynamics have been assessed in only a few species, which does not allow for generalization of findings across bat taxa and geographic regions.

**Methods:**

To assess the generalizability of coronavirus shedding seasonality, we sampled hundreds of bats belonging to several species with different life history traits across East Africa at different times of the year. We assessed, via Bayesian modeling, the hypothesis that chiropterans, across species and spatial domains, experience seasonal trends in coronavirus shedding as a function of the reproductive cycle.

**Results:**

We found that, beyond spatial, taxonomic, and life history differences, coronavirus shedding is more expected when pups are becoming independent from the dam and that juvenile bats are prone to shed these viruses.

**Conclusions:**

These findings could guide policy aimed at the prevention of spillover in limited-resource settings, where longitudinal surveillance is not feasible, by identifying high-risk periods for coronavirus shedding. In these periods, contact with bats should be avoided (for example, by impeding or forbidding people access to caves). Our proposed strategy provides an alternative to culling – an ethically questionable practice that may result in higher pathogen levels – and supports the conservation of bats and the delivery of their key ecosystem services.

## Background

The order Chiroptera is the second largest order of mammals with more than 1000 identified species [1]. The members of this order, bats, provide important ecosystem services (reviewed in [2, 3]). For example, insectivorous bats can reduce arthropod herbivory [4–6], increase agricultural yields [7], reduce the need for insecticides [8], and prevent large financial losses in agriculture [9–11]. Plant-visiting chiropterans provide pollination and seed-dispersing services (reviewed in [3]), certain nectivorous bats are pollinators of economically important plants [12], and frugivorous bats can be important for reforestation [13]. Finally, cave-roosting bats produce guano, the main energy source in many cave ecosystems [14, 15], and the mining of this product is an income source in poor communities [16].

However, current evidence supports that bats are a natural host of several disease-causing viruses across the globe, including zoonotic viruses, such as rabies virus (Rhabdoviridae, genus *Lyssavirus*); Hendra and Nipah viruses (Paramyxoviridae, genus *Henipavirus*); and Marburg and ebolaviruses (Filoviridae, genus *Marburgvirus* and *Ebolavirus*, respectively; [17, 18]). Bats are also hosts of several viruses of the *Coronaviridae* family [19–22]. Molecular evidence suggests that the Severe Acute Respiratory Syndrome *Betacoronavirus* (SARS-CoV betaCoV) and the Middle-East Respiratory Syndrome betaCoV (MERS-CoV) originated from bats [23–31]. Both viruses emerged in humans during the past two decades, specifically in China (2002) and Saudi Arabia (2012). The SARS-CoV pandemic included 8096 cases in 27 countries with a ~ 10% case fatality [32], while MERS-CoV has affected 2279 people in 27 countries with a case fatality of ~ 35% [33]. Incidental cases of MERS-CoV are still detected mainly in Saudi Arabia [32], and it is thought that camels are important for human infection [34–36].

Today it is known that: i) a high genetic diversity of coronaviruses (CoVs) is present in more than 100 bat species, including viruses related to SARS and MERS CoV [37]; ii) CoVs are prone to move and adapt to new host species [38]; iii) plausibly, all mammalian-adapted CoVs may have originated in bats [20, 21, 38], including a recently emerged highly fatal *Alphacoronavirus* in piglets [39] and the 229E human CoV [22, 40, 41]; and iv) CoVs found in bats can use human receptors for cell entry [21, 25, 42, 43]. These lines of evidence suggest that future spillover of coronaviruses humans is feasible.

Because CoVs are found in bat species that have adapted to be in close contact with humans, such as the straw-colored fruit bat (*Eidolon helvum*) and the Brazilian free-tailed bat (*Tadarida brasiliensis* [44, 45]), high contact “bat-human” interfaces currently exist around the world. If the bats in these interfaces shed CoVs with the ability to infect humans, then opportunities for spillover through direct exposure to feces [37] or the contamination of food are created, as these viruses can remain infectious in the environment for days [46]. Therefore, strategies aiming to mitigate human exposure to CoVs, and thus, the risk of spillover and disease emergence are needed, while supporting the conservation of bats and their important ecological roles.

Longitudinal sampling with specific species has shown that the proportion of bats shedding CoVs varies seasonally [47–50] and that fecal CoV-RNA loads can also be heterogeneous over time [51, 52]. If exposure through contact with bat feces is a main pathway for zoonotic CoV spillover to humans but shedding of these pathogens is not uniform over time, then mitigation strategies aiming to prevent bat CoV-shed exposure could be targeted temporally, directed especially at high-risk seasons. Such a strategy could guide policy in limited-resource settings where sampling bats for CoV testing is not feasible and it could support an ethically acceptable management to mitigate spillover risk. However, the few species and locations tested to date do not allow for identification of a potential seasonal shedding pattern to responsibly suggest temporal spillover risk management across species and geography. Therefore, assessment of the CoV dynamics in a broader range of bat species that show different life history traits, as well as in diverse geographic and ecological circumstances, could be extremely useful.

To this end, we evaluated the dynamics of CoV shedding in different bat species sampled in several locations in East Africa at different times of the year. This geographical region has been identified as a hotspot of pathogen emergence [53], where CoV host switching events seem to be higher compared to other areas [22], but, to our knowledge, no study on CoV dynamics in bats has been conducted. Specifically, we hypothesized that bat species exhibit seasonal trends in CoV shedding that are associated with the reproductive season. We assessed this hypothesis by fitting Bayesian statistical multivariable models to evaluate whether CoV shedding in bats is positively associated with the time period when pups are becoming independent from the dam. Beyond the inclusion of several species sampled in different countries at different times, we explicitly identified the reproductive events for each species at the time of sampling and also included other traits, such as the aggregation of individuals at the roost, that may be involved in CoV dynamics.

## Methods

### Sample collection

**Fig. 1:**
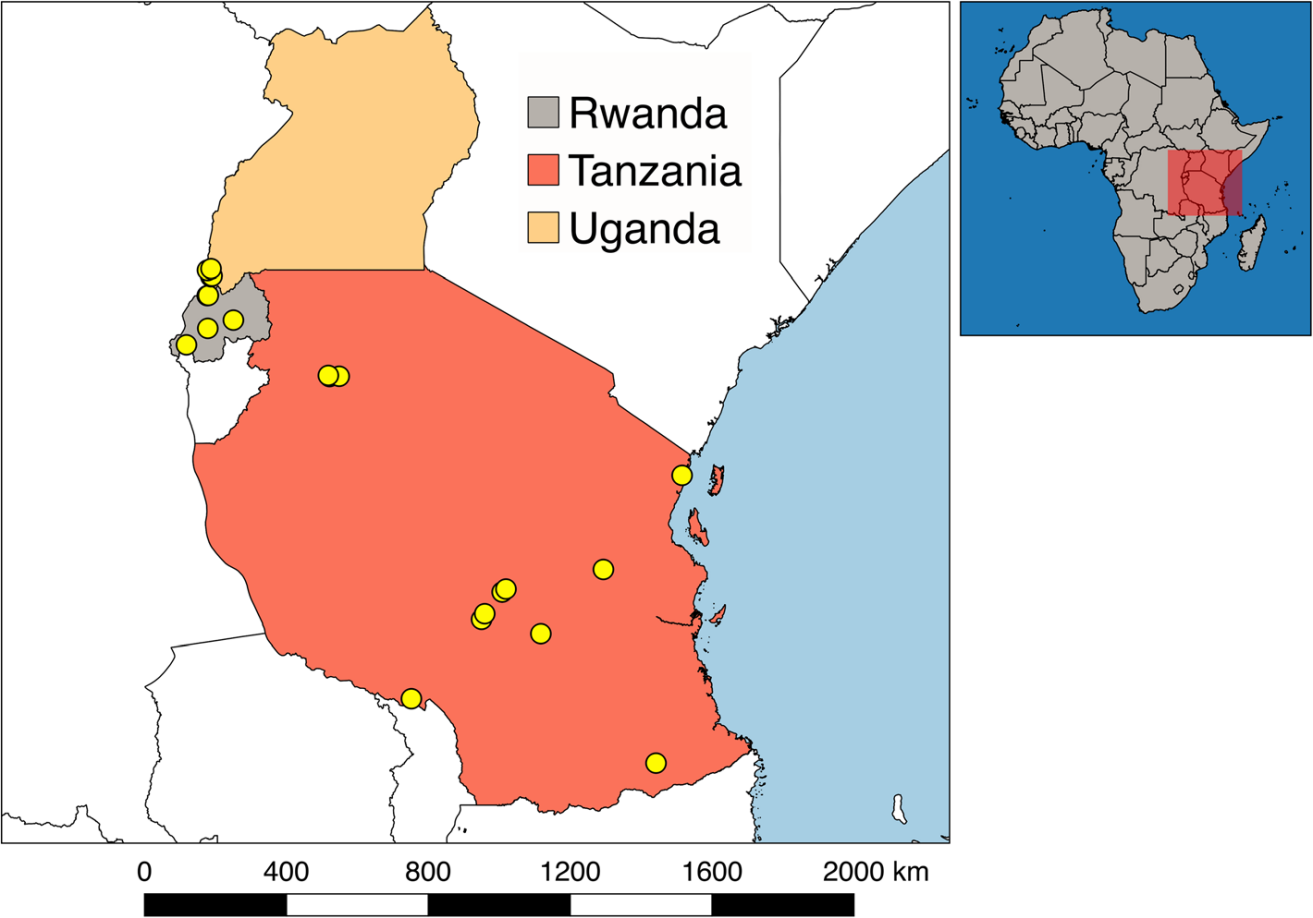
Unique locations where samples from bats were obtained

Samples (rectal swabs and fresh feces) were collected from bats captured in Uganda, Rwanda, and Tanzania (Fig. 1), between September 2011 and April 2014 with permission from local authorities and under the Institutional Animal Care and Use Committee at the University of California, Davis (protocol number: 16048).

Bats were captured in 36 unique locations between latitudes − 0.9739 and − 10.7506 (Fig. 1). These locations were selected because they represented potentially high-risk interfaces for contact between bats and humans, such as areas of land-use change, human-dwellings, ecotourism sites, markets, and places with potential for occupational exposure [54]. Locations in close proximity (Euclidean distance < 20 km) in which sampling was conducted within the same week were considered a single sampling event. The remaining sampling events that occurred in the same location or spatially close to others but conducted in different weeks were considered independent sampling events. As result, we collected samples from 30 unique sampling events.

All captures were conducted using mist nets set in the early morning or at dawn. Individuals were released after sample collection. Samples were handled as previously described [22, 55]. Each sample was immediately transferred to vials containing Viral Transport Media and NucliSens® Lysis Buffer (bioMérieux, Inc., Marcy-I’Étoile, France), which were maintained in liquid nitrogen until storage in a -80C freezer in each country.

### Coronavirus detection

RNA was extracted from all samples, and cDNA was prepared as previously described [22, 55]. Two broadly reactive consensus PCR assays targeting non-overlapping fragments of the orf1ab were used to detect both known and novel CoVs [56, 57]. Amplified products of the expected size were cloned and sequenced as described in [22]. Sequences were analyzed and edited using Geneious Prime 2019.1.3 [58].

A sample was considered positive when at least one PCR assay yielded a sequence that matched coronaviruses in GenBank. Coronavirus sequences were classified as belonging to a specific taxa following previously described methodologies [22].

### Bats age, sex, and species identification

Bats were categorized as adults and juveniles based on size, and morphological and behavioral characteristics were observed at sample collection. The sex of the bats was also recorded.

Identification of some bat species can be challenging in the field. For this reason, field-identified species were confirmed by DNA barcoding using the cytochrome b or cytochrome oxidase subunit 1 mitochondrial genes [59]. Obtained sequences were compared against sequences in the GenBank and Barcode of Life databases [60, 61]. When possible or necessary, sequences from both genes were used for species identification. A threshold of 97% nucleotide identity was used to confirm the species; sequences with 95–97% nucleotide identity were assigned a “conferre” (cf.) species status, and sequences below 95% nucleotide identity were either classified to the genus level or as unidentified. Sequences with > 97% nucleotide identity to more than one species for either gene, were classified to the genus level unless they clearly clustered with sequences from other animals in the same geographic area.

If barcoding results for all of the first ten bats tested per species were in agreement with the field identification, we assumed that the field identification for the remaining bats of that species in each country was correct. Otherwise, all of the remaining samples were barcoded to ensure correct speciation.

### Bat life history

We recorded when sampled females were pregnant based on abdominal palpation, had attached pups (indicating recent parturition), and were lactating, as well as when juveniles were captured. Therefore, we were able to track pregnancy, lactation, and recent birth pulses. Moreover, we accessed the data in the PanTHERIA [62] and Amniote [63] databases, and we thoroughly reviewed the literature on the biology of the bats species we sampled for latitudes similar to our sampling locations. With the gathering of these information sources we established the timing of the birth pulses, lactation periods, and the weaning of pups for each species. For the details justifying the dates inferred for these three life history events for each species and the corresponding bibliographical references see Additional file 1.

Once the timing of these events was confirmed or inferred, we were able to establish 2 seasons that occur at least once during the year across all observed bat species: i) when juveniles are being weaned and female-pup contact decreases (“Recent weaning” [RW]) and ii) the rest of the year (hereafter “N-RW” for “Not recent weaning”). We chose to evaluate risk of CoV shedding for the first period because past longitudinal studies with microchiropterans in Germany and China found higher CoV-RNA loads approximately 1 month after parturition [51, 52]. Similarly, peaks were found 2 months after the formation of a maternity colony of *Myotis macropus* [49], which would match a post weaning period for that species. Here, we defined the end of the RW period as 1 month after the last pups were weaned. We assumed that 1 month would provide a reasonable time window for the colony to “clear” the CoV susceptibility status of this period and acquire the susceptibility corresponding to the season(s) when weaning does not occur (N-RW period), if differences actually exist.

Finally, once we determined these two seasons, we categorized each bat sample into one of them depending on the week of the year in which they were taken. Because some species had more than one litter per year, there could be more than one RW period during the year. It is worth noting that we were able to define these periods for those species in our dataset that have synchronized reproduction, whose biology was properly described, and whose taxonomy is generally accepted. When we could not assign a reproductive period to specific bats, this season was imputed (see Methods: Statistical Analysis).

### Bat species traits

We characterized specific traits of each bat species studied based on previous scientific literature on pathogen dynamics in bats [51, 64–71]: colony size (small, medium, or large if a typical colony contains one to dozens, hundreds to thousands, or thousands to millions of individuals, respectively); roost type (“closed” if the species has been reported to use caves, mines, roofs, or other confined spaces; or “open” if the bats have been typically reported roosting in the foliage of trees); the aggregation of bats in clusters while roosting (no, yes); and the number of litters per year of the species at equatorial latitudes. References are provided in Additional file 2. We also considered data from PanTHERIA and Amniote [62, 63].

We could not include other species traits, such as the mating strategy (harem or another) and the segregation of females in maternity colonies, because available studies were incomplete or contradictory. We did not include factors, such as multi-species occupancy of the roost, because we did not observe all of the roosts. Further, we did not assess postpartum estrus, as within the study area, it is only known to occur in some Molossid bats [72], of which we only sampled a small number.

### Statistical analysis

To statistically estimate the association between the RW and CoV detection we used a Bayesian inference to model the detection of CoVs as a *Bernoulli* process of the form:

$$ Co{V}_i\sim Bernoulli\left({p}_i\right)\  withi=1,\dots, I $$

where CoV_*i*_, the detection of CoV in rectal swabs (1 if detected, 0 otherwise) from the *i*^th^ bat (sample), is assumed to follow a *Bernoulli* process parameterized by *p*_*i*_, the probability of CoV detection on the *i*^th^ bat. This parameter was related to a set of candidate covariates as:

1 $$ logit\left({p}_i\right)={\alpha}_0+{\beta}_1{X}_{1i}+\gamma {Y}_i+{\rho}_1{C}_{1i}+\dots +{\rho}_l{C}_{li}+{S}_{j(i)}+S{p}_{k(i)}, $$

with *S*_*j*_~*Normal* (0, *σ*_*S*_) *and Sp*_*k*_~*Normal* (0, *σ*_*Sp*_).

Here X and Y are binary covariates representing the RW season and juvenile age category. We specifically included these two terms to separate the potential association of the season with CoV detection from the seasonal presence of juveniles. Because it was not feasible to allocate all species in the RW or N-RW seasons based on previous research, we assumed that these unknown reproductive seasons were “Missing at Random” and they were imputed as a function of the latitude at sampling, the day of the year of the sampling event, the number of litters per year of the corresponding species (one litter per year versus more than one litter per year), and the historical precipitation of the month at the sampling event location. The description of the imputation model is provided in Additional file 3.

The terms *S*_*j*_ and *Sp*_*k*_ represent the sampling event- and the species-specific intercepts, respectively, because we assumed that bats sampled in the same event and bats belonging to the same species were not independent with respect to CoV detection. No sampling event involved the same bat colony in successive RW and N-RW seasons, therefore, we assumed that CoV detection was not temporally correlated within sampling locations.

We constructed the model by adding other covariates one-at-a-time to this working model: the C_1*...l*_ categorical variables; and they remained in the model if they were judged to confound the relationship between CoV detection and the reproductive seasons or between age and CoV detection (i.e., their inclusion caused meaningful changes in the Posterior Probability Distributions [PPDs] of the specific reproductive season or age coefficients). Finally, C_1*...l*_ categorical variables could be retained as well if they were marginally associated with CoV detection (the corresponding coefficient PPD did not include zero). To assess the goodness-of-fit of the models, we evaluated the congruence between CoV detection in the data and in the posterior predictive distributions yielded by the models by: i) ages and seasons, ii) age, iii) per season, iv) per age and season, and v) per sampling event.

All models were constructed using “Stan” v. 2.17.0 [73] which was run from “R” v. 3.6.0 [74] through the package “RStan” v. 2.17.3 [75]. Weakly informative priors were assigned for all coefficients: Normal(0, 1.5) for the estimates of α_0_, β’s, γ, and ρ’s. The σ_S_ and σ_Sp_ had a prior Half-Cauchy (0,5) following previous suggestions [76, 77]. The PPDs were estimated by sampling in parallel from 4 MCMC chains for 4000 iterations following 3000 iteration warm-up for a total of 4000 saved samples for each parameter PPD. Convergence was assessed by the Gelman-Rubin statistic [78] and graphically using trace plots. The code to fit the models is available at https://github.com/dmontecino/East-African-bats-and-CoV-shedding.

## Results

### Bat samples, age, sex and reproductive seasons

**Fig. 2:**
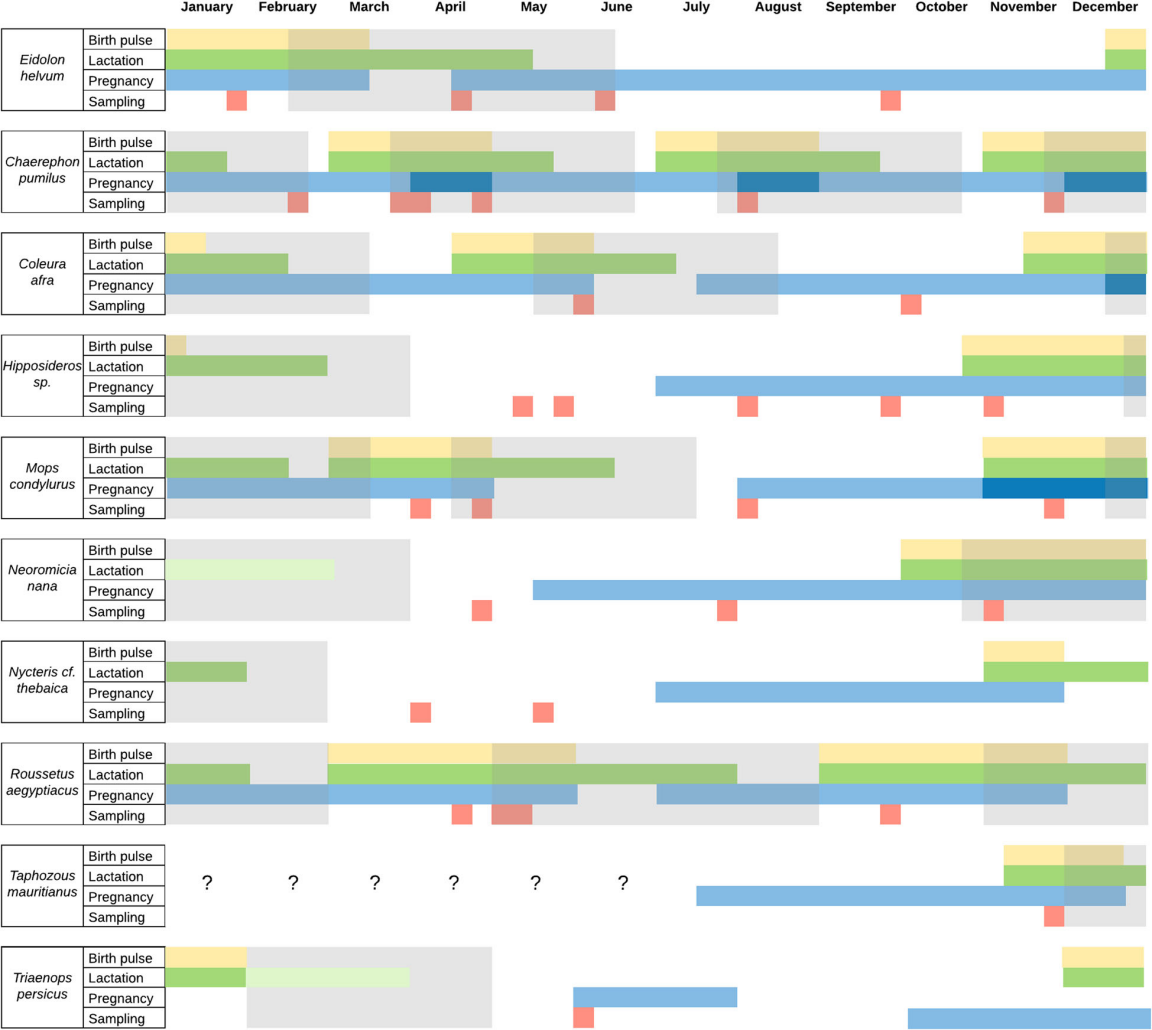
Summary of the inferred reproductive periods of the bat species sampled. The red squares show the week of a typical year each bat species was sampled. The yellow, green, and blue polygons show the assigned extent of the birth pulse (yellow), lactation (green), and mating-pregnancy periods (blue). The grey polygons represent the inferred “Recent weaning” period(s) per bat species. In species with unknown lactation length (*N. nana* and *T. persicus*), the light green polygons represent the likely overextension of this period not including any other bat in the “Recent weaning” period. The question marks show the period we could not infer the corresponding reproductive activities

We sampled 753 individuals, all of them aged and successfully identified to belonging to 15 species. *Nycteris thebaica*, *Pipistrellus hesperidus*, and *Rhinolophus clivosus* were assigned the “*conferre*” status. Hipposiderids were assigned only to the genus level because barcoding did not provide certainty on species identification in line with previously recognized taxonomic difficulties [79–81]; however, the biology of the candidate hipposiderid species is similar (Fig. 2; [82, 83]), and we were able to used them for estimation purposes. We excluded *Scotophilus viridis* (*n* = 6) from the analysis because their reproductive traits are unknown, and this species also has taxonomic difficulties for species identification [84]. Therefore, these six individuals were removed, and 747 bats were included in the study.

We were able to infer the reproductive season for all bats except for *Lissonycteris angolensis*, *Rhinolophus* cf. *clivosus*, and *Pipistrellus* cf. *hesperidus* (*n* = 117, 104 adults and 13 juveniles) that had limited available knowledge on biology and reproductive season. These reproductive seasons were imputed as explained above. This imputation process did not substantively affect the proportion of bats in each reproductive period or the crude CoV detection per age (Additional file 3: Figure S3.2). Moreover, we had partial data for *Neoromicia nana* and *Triaenops persicus* lactation period and we assigned one that is likely longer than it would be in reality based on the other species. Even under this overextended period, none of these bats (*N. nana* and *T. persicus*) could have being sampled during the RW season, so this knowledge gap did not risk a misclassification (Fig. 2).

In the end, 274 and 356 bats were allocated in the N-RW and RW periods, respectively. The distribution of bats across the reproductive periods per age and sampling event was heterogeneous as expected due to the opportunistically nature of sampling. Indeed, 233 and 273 adult bats were in the N-RW and RW periods, respectively, while 41 and 83 juvenile bats were sampled in these seasons, respectively.

### Coronavirus detection and identification

In total, 30.79% bats were positive for CoVs (*n* = 230). Within the subset of adult bats, 26.22% were positive (*n* = 160) while 51.09% of the juvenile bats were positive (*n* = 70). The detection of CoV shedding was variable across seasons and bat species, as well as across sampling events (0, 100, 9.69, and 25.84% for the minimum, maximum, median, and mean detection, respectively). The CoVs found per species are shown in Additional file 4: Table S4.

### Species traits

A summary of the roosting and reproductive traits of the bat species sampled is provided in Table 1. All bats except *N. nana* (*n* = 9) and *E. helvum* (*n* = 315) roosted in “closed” structures, such as caves, abandoned mines, and roofs. Within the group of bats using “open” structures, *E. helvum* was the only species with CoV positive individuals. Therefore, we did not use this covariate to assess a potential association with CoV shedding.

**Table 1:** Summary of traits by sampled chiropteran species

Chiropteran species	Trait			
	Colony size	Roost type	Aggregation in clusters while roosting	Number of litters per year
*Chaerephon pumilus*	Medium	Closed	No	3^b^
*Coleura afra*	Large	Closed	No	2
*Eidolon helvum*	Large	Open	Yes	1
*Hipposideros sp.*	Medium	Closed	Yes	1
*Lissonycteris angolensis*	Medium	Closed	No	2
*Mop condylurus*	Medium	Closed	Yes	2
*Neoromicia nana*	Small	Open^a^	No	1
*Nycteris* cf. *thebaica*	Medium	Closed	No	1
*Pipistrellus* cf. *hesperidus*	Small	Closed	Yes	1
*Rhinolophus* cf. *clivosus*	Small	Closed	No	1
*Rousettus aegyptiacus*	Large	Closed	Yes	2
*Taphozous mauritanus*	Small	Closed	No	2
*Triaenops persicus*	Large	Closed	Yes	1

^a^ *Neoromicia nanus* roosts in folded banana leaves. Could be considered “Closed” also

^b^ It has been proposed up to 5

### Statistical analysis

The models showed adequate sampling. The 4 Markov Chain Monte Carlo chains converged graphically, whilst all Gelman-Rubin statistics were < 1.004. The selected model had a number of effective samples for each coefficient of at least 1636. The data were properly fitted, as well (Additional file 5: Figure S5.1), although some predictions lacked precision. The PPDs of the fixed coefficients are shown in Additional file 5: Figure S5.2.

The selected model to assess periodic differences in CoV shedding included season and age, species-specific intercepts, and sampling event-specific intercepts. Beyond the species-specific terms, we included a binary categorical covariate equal to 1 for *E. helvum* and *T. persicus* and 0 otherwise. We incorporated this term because CoV detection in these species was remarkably higher than the other species. As expected, this fixed effect was correlated with the corresponding species-specific intercepts (the remaining correlations were all low); however, we decided to keep it to assess if the main findings hold even when accounting for the bat species with highest detection. The corresponding means, standard deviations, and 90% HPDI are shown in Table 2.

**Table 2:** Summary of the posterior probability distributions of the fixed-effects coefficients of the selected model

Covariate	Mean	SD	90% HPDI
Intercept (α_0_)	−3.21	0.77	−4.375 - -1.944
Recent weaning	1.62	0.68	0.538–2.772
Juvenile age class	0.66	0.26	0.233–1.078
*E. helvum – T. persicus*	1.33	1.17	−0.511-3.282

*SD* Standard deviation and 80%, *HPDI* = 90% high posterior density interval

**Fig. 3:**
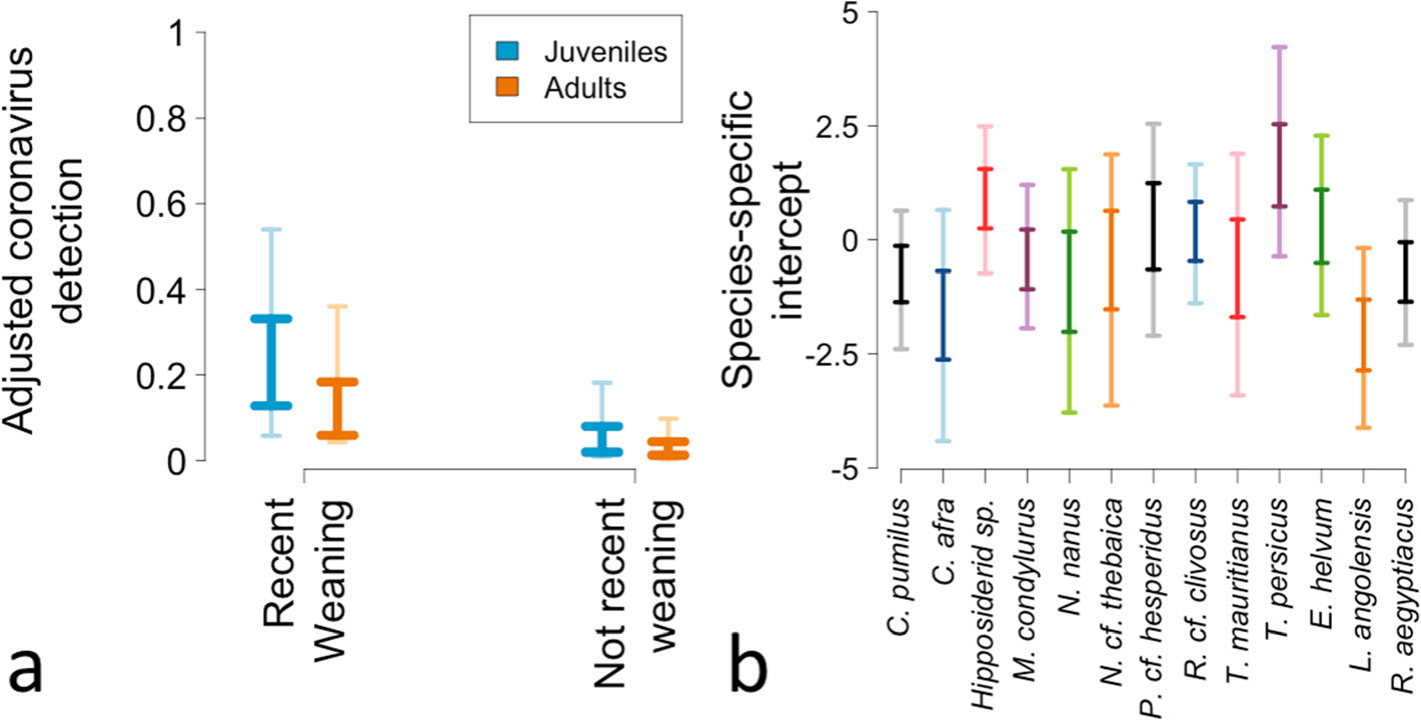
**a** Modeled proportion of chiropterans shedding coronavirus viral particles in two reproductive periods inferred for these bats by age class. **b** The estimated species-specific intercept coefficients. The lighter colors represent the range where the 90% of the estimated detection values are concentrated (the High Posterior Density Interval). The darker colors show the 51% High Posterior Density Interval

The coefficients’ PPDs from the selected model indicate an association between age and CoV shedding, with juveniles presenting 1.26–2.94 times higher odds to shed compared to adult bats (90% HPDI). The coefficients’ PPDs also point to an association between the reproductive season and CoV shedding as well, with an estimated odds 1.71–16.00 times higher to shed during the period when pups are being compared to other seasons (90% HPDI). The proportions of CoV shedders estimated by reversing the 90% HPDI of the logits were: 0.02–0.22, 0.09–0.59, 0.01–0.13, and 0.05–0.42 for juveniles during the “N-RW” and “RW” periods, and adults during the “N-RW” and “RW” periods, respectively (90% HPDI). These values refer to bats not belonging to the species *E. helvum* or *T. persicus*. Finally, the predicted CoV detections, based on the posterior predictive distributions, were 0.01–0.18, 0.06–0.54, 0.00–0.05, and 0.04–0.36, for these same groups (90% HPDI; Fig. 3, left). In practical terms, these last values imply that juveniles during the “RW” period are, on average, 3.34 times more likely to be detected shedding CoVs compared to juveniles in the “N-RW” period. Adults during the “RW” period are, on average, 3.93 times more likely to be detected shedding CoVs compared to adults in the “N-RW” period. In both seasons, juveniles are, on average, more likely to shed CoVs, than adults.

The selected model suggests a higher odds of CoV detection in *E. helvum* and *T. persicus* compared to other species. The species-specific intercept terms, once the *E. helvum* - *T. persicus* effect is included, suggest no further differences in terms of CoV shedding (Fig. 3, right); however, the estimates are not precise. The sampling event-specific random intercepts suggest that a few specific locations could show differential CoV shedding but that most of them do not explain further variation (Additional file 6).

## Discussion

If coronavirus shedding by bats follows temporal patterns that are generalizable across species and locations, then mitigation strategies targeting the prevention of human exposure and potential spillover could be directed toward high-risk periods, through mechanisms that can also support bat-human coexistence and the provision of bat ecosystem services. Previous research has focused on viral identification in specific locations and in few species [47–50], resulting in a limited representation of viral dynamics in association with few ecological settings, biological traits, and reproductive strategies. Additionally, few studies of coronavirus shedding patterns have employed statistical models and, in consequence, the potential complex web of factors and causal relationships that may determine this process has not been fully explored. Here, we aimed to address these issues by statistically modeling coronavirus RNA detection in several bat species, captured at different times and locations in East Africa and involving different ecological contexts and life histories. Using data from several hundreds of bats, we found that, beyond spatial, taxonomic, and life history differences; i) the odds of coronavirus shedding is higher during the period when pups are being weaned (up to a month after the lactation period is over), and ii) juvenile bats have higher odds to shed these viruses. Moreover, the ratios of predicted detections per bat category (age and reproductive season) suggest that juvenile bats during the recent weaning period have relatively higher shedding compared to bats out of this period no matter their age. Caution must be taken with these ratios because we used a logit link and our data had high proportion of CoV shedding in specific groups. However, our results are consistent, and they are in agreement with previous research conducted in a restricted number of species and locations.

Similar seasonality of coronavirus shedding has been observed in Germany, Australia, Thailand, China, and Ghana (West Africa). In the specific species involved in these previous studies, higher coronavirus shedding and viral loads were detected weeks after the birth pulse [47–52, 85]. Further, and consistent with our results, detection of higher levels of coronavirus in juveniles has been reported in micro- as well megabats from Africa, Asia, Europe, and North and South America [22, 48, 50, 86–89].

It has been proposed that the increased detection of coronaviruses after the birth pulse is attributable to the waning of passively-received maternal antibodies in juveniles [51]. This idea has been frequently cited; however, we are not aware of any longitudinal age-specific coronavirus seroprevalence study in bats. Such studies are important to understand the drivers of pathogen persistence and spillover risk, and in consequence, to ethically manage and prevent bat pathogen exposure. Nevertheless, this kind of research is difficult to conduct due to logistical challenges, our questionable ability to obtain statistically-representative samples across age groups, cross-reactivity of serological assays, and the difficulties to differentiate serodynamics derived from closed-population processes from those caused by migratory movements. Although extrapolations for antibody dynamics across viruses and species are not simple [90], bat serodynamics for Hendra virus are congruent with the increased detection of coronaviruses after the birth pulse. Pups passively receive maternal Hendra virus antibodies which decline after the first month of age up to 6 months of age [91–94]. This decline would lead to a period in which young bats tend to be more susceptible to infection, become infected, and then shed virus. Consistently, coronavirus shedding peaked weeks after the birth pulse in a German and a Chinese species [51, 52] and immunologically naïve bats shed higher coronavirus loads [30]. Over time, as young bats clear Hendra virus infection, they become seropositive again [91–94]. Concordantly, capture-mark-recapture studies support the clearance of coronaviruses in infected bats [47, 95], which would become seropositive. However, young bats may not reach adult seroprevalence levels until they are older than a year, as occurs with Hendra virus [91, 92, 94]. Therefore, the population of juvenile bats would remain comparatively more susceptible to viral infection and shedding beyond the period immediately after weaning. Age-specific henipavirus seroprevalence in African *E. helvum* is in agreement with the serodynamics described for Hendra virus [90].

Additionally, coronavirus transmission may be favored by high colony density created by the birth pulse, as previously proposed [85, 95], and then the seasonal influx of susceptible juveniles could accelerate viral spread across the entire colony, including adult bats. Indeed, adult *Myotis macropus* in an Australian colony showed a peak of coronavirus detection after the birth pulse [85]. The peak of coronavirus detection for two *Hipposideros* species and *Nycteris* cf. *gambiensis* sampled in Ghana occurred during the months that encompassed the birth pulse and nursing after accounting for the age of the sampled individuals [50]. Higher coronavirus infection has also been reported in lactating females [85, 88], which overlap with the period of pup weaning and decay of maternally-derived immunity; however, the opposite has also been found [48, 50, 85].

In practical terms, public health managers could anticipate high risk periods for coronavirus shedding to target interventions. Assuming that higher spillover risk is a function of higher viral shedding [67] and that all coronaviruses with zoonotic potential behave ecologically similarly to coronaviruses detected in this study, managers could target the prevention of human-bat direct (consumption) or indirect (bat droppings) contact specifically during the high-risk season: around and just after weaning, the timing of observable juveniles or individuals smaller than adults. For the species and interfaces defined herein, those management periods have now been determined (Fig. 2). For others, direct observation of bats at high-risk transmission interfaces could be used to identify time periods when non-adult sized bats are present. However, observation of dependent pups is not always easy [96]. Of course, for specific species, birth pulses and lactation seasons could also be used to more precisely establish high risk periods similar to the methods we used here, including a combination of direct observation, reports from previous literature, and consultation with knowledgeable bat biologists. Our proposed risk-driven strategy i) is evidence-based, as it builds upon coronavirus shedding patterns observed across several chiropteran species present around the world; ii) does not require the advanced laboratory capacity often lacking in resource-restricted settings where intense bat-human interfaces usually occur; iii) is a good alternative to the ideal but expensive and resource-intensive longitudinal surveys; and iv) it may prevent the exposure to viruses belonging to other taxa whose observed bat shedding dynamics resemble our findings for coronaviruses (e.g. paramyxoviruses [97]),

The lower coronavirus detections in African emballonurids (*C. afra* and *T. mauritianus*) and the higher detections in *E. helvum*, African hipposiderids (*Hipposideros sp*. and *T. persicus*), and *R.* cf. *clivosus* that we found are consistent with previous reports [22, 44, 50, 98] and should be considered by managers when providing risk-based spillover prevention strategies. Moreover, SARS-like coronaviruses in Africa have been found in hipposiderid, rhinolophid, and molossid bats [22, 57, 98], and MERS-related coronaviruses have been found in vespertillionid bats [22, 28, 29]. Therefore, it seems reasonable to prioritize the identification of birth pulses and lactation seasons, and thus determine high-risk periods of coronavirus shedding, for these bat families. Interestingly, *E. helvum* roost in tight clusters that can contain hundreds of individuals [99], similar to *T. persicus*. On the other hand, emballonurids, showing the lowest crude detection levels across families (represented by *Coleura afra* and *Taphozous mauritanus* here) tend not to cluster while roosting [100, 101]. We did find an association between coronavirus shedding and whether the species typically aggregate in clusters while roosting when the variable “*E. helvum* – *T. persicus*” was not considered, but we chose a different model not including this term because we did not directly assess bat roosts and our categorization may oversimplify the continuum from mostly solitary roosting (e.g., *Neromocia nana*) to common tight aggregations of bats (e.g., *Mops condylurus*). Using this categorization could be misleading, as some species differentially cluster while roosting depending on temperature, colony size, colony type (e.g., maternity colony versus not a maternity colony), and season [72]. Future studies should consider the roosting habits of bats, as this trait could further support risk-based management to prevent or reduce human exposure.

The risk-driven strategy we propose provides a contact-reduction alternative that is ethically favorable compared to often-employed measures, such as culling or other reactive measures, that ensue when the public becomes aware of a health threat without a suggested practical option to reduce their risk for exposure. In addition to ethical concerns and being logistically difficult and expensive, culling has failed to reduce disease in wild populations and can result in even higher pathogen levels. For example, “badger culling can make no meaningful contribution to cattle tuberculosis control in Britain” [102]. A culling program to reduce *Echinococcus multilocularis* prevalence in red foxes (*Vulpes vulpes*) resulted in an increase of infection [103]. This strategy has also failed to control rabies in canids around the globe [104]. Similar results have been observed in bats. Culling failed to reduce rabies seroprevalence in *Desmodus rotundus* in Perú and could have increased the levels of exposure to the virus [105, 106]. In Argentina, the extermination of bats changed the direction of spread of rabies in livestock but did not prevent its advancement [107]. In Uganda, miners exterminated a colony of *Rousettus aegyptiacus* bats after an outbreak of Marburg virus in 2007 that involved 4 miners in close contact with these bats. Five years later, a new outbreak occurred in miners from the same mine. The second time, Marburg virus RNA was detected in a higher proportion in the *R. aegyptiacus* that recolonized the mine (13.3%, *n* = 400; [108]) compared to RNA detection before culling in this cave (5.1%, *n* = 611; [109]) and other caves in Uganda (2.5%, *n* = 1622; [67]) and Gabon (4.8%, *n* = 187; [110]) where culling has never been reported.

Culling can also cause demographic changes, leading to a higher proportion of juvenile individuals. This change may occur because of a disproportionate cull of older individuals; the potential increase in survival of pups at lower population densities, followed by higher recruitment of juvenile females into reproductive age [111]; the hypothetical increase of young dispersers immigrating from neighboring colonies into culled, less dense, and better resourced colonies [105, 112]; or by causing compensatory reproduction [113]. This last possibility may have not been studied in bats but seems unlikely due to their high conception rates and usual litter size of one. Examples of younger populations after culling have been reported in the red deer (*Cervus elaphus*), racoon (*Procyon lotor*), American mink (*Mustela vison*), and Australian brushtail possums (*Trichosurus vulpecula*), among others [114–117]. As our results and past research consistently show higher viral shedding and detection in young individuals, activities leading to a younger bat population are not advisable for viral spillover management. Similar results are expected when fruit bats are culled based on being categorized as “agricultural pests”; therefore, this kind of management may create higher risk of viral exposure to the human population.

Virological, ecological, and epidemiological research on bats over the last 15 years has helped to identify chiropterans as hosts of zoonotic viruses and to document that human-driven environmental change, human behavior, and human-to-human transmission are the key drivers for the creation of bat-human interfaces, spillover, and epidemics of emergent viruses, respectively [118–120]. In the context of the current biodiversity and bat conservation crisis [121, 122], we must not omit these facts when attempting to effectively, and responsibly frame and communicate disease risks associated with bats. Realistic, data-based risk communication is of paramount importance to avoid framing bats as a threat to humans and to support bat conservation given their important ecological roles [123, 124]. With this background, it seems a proper time for the scientific community studying “bat-associated” viruses to move the conversation from bat spillover risk assessments to the planning of pro-biodiversity and subsequently pro-ecosystem strategies aiming to mitigate spillover risk. Science is valued not only for the diagnosis of problems but because it finds solutions to them. Here, we have attempted to aid the progress of scientific and management dialogue by proposing, not only a management strategy to limit potential coronavirus spillover, but one that is context- and logistically-grounded and pro-conservation, promoting the delivery of the key ecosystem services provided by bats.

## Conclusions

Data from hundreds of bats collected in East-Africa show that coronavirus shedding is expected to be more frequent when pups are becoming independent from the dam, independently of the age of the bats, their species, their location, and their life histories; however, the odds of shedding do differ by species. These results can guide temporal-based mitigation strategies to prevent bat-associated coronavirus exposure using non-lethal methods in limited-resource settings, where longitudinal surveillance is not feasible, by identifying high-risk periods for coronavirus shedding when contact with bats should be avoided.

## Supplementary information

**Additional file 1:** Summary of the inferred start date of the birth pulse, the end of the lactation period, and the start date of the mating period per microchiropteran species.

**Additional file 2:** Bibliographic references for the traits of the bat species included in the study.

**Additional file 3:** Imputation of the reproductive season to those bats whose biology is insufficiently known. Model methods, results, and literature cited. **Table S3**. Summary of the coefficients’ posterior probability distributions of the selected model for the imputation of the reproductive seasons of bats missing this data. **Figure S3.1**. The discrepancy between the observed (inferred) period and the period predicted by the selected imputation model considering 5 thousand posterior predictions of the observed period. Values of zero indicate matching, (observed period - predicted period). **Figure S3.2**. Left: the proportion of bats in each period after the imputation for those bats with the non-inferred season. Each line connects the proportion of individuals per reproductive season in each of the 5,000 Markov Chain Monte Carlo iteration. Right: the distribution of the crude coronavirus detection per reproductive season across the 5,000 Markov Chain Monte Carlo sampling iterations after imputing the periods when un-inferred. The black boxplots show the distribution of the coronavirus detection per period, while the light and dark colored boxes above and below show the interquartile detection in non-adults and adult bats, respectively, per period.

**Additional file 4: Table S4.** Summary of the Alpha- and Betacoronaviruses (alphaCoV and betaCoV, respectively) found in the microbats tested.

**Additional file 5:** Results of the model to assess the association between CoV shedding and the “Recent weaning” season in *Eidolon helvum* and microbats. **Figure S5.1**. Posterior predictive distributions of coronavirus positive bats (histograms) and the observed coronavirus positive bats (vertical lines). The histograms show the distribution of four thousand predictions of detection in the sampled bats. The lines show the observed detection. A) Coronavirus detection across all bats. B) Coronavirus detection across age categories: adults (light blue) and non-adults (yellow). C) Coronavirus detection across the reproductive seasons: “Not recent weaning” (light blue) and “Recent weaning” (yellow). D) Coronavirus detection across the reproductive seasons in the non-adult bats: “Not recent weaning” (light blue) and “Recent weaning” (yellow). E) Coronavirus detection across the life history seasons in the adult individuals: “Not recent weaning” (light blue) and “Recent weaning” (yellow). **Figure S5.2**. The density of the coefficients’ posterior probability distributions of the selected model. RW refers to the “Recent weaning” period (versus “Not recent weaning” season).

**Additional file 6:** Sampling events -specific random intercepts values.

## Data Availability

The datasets used and/or analyzed are available at 10.6084/m9.figshare.9917762.

## Abbreviations

betaCoV: *Betacoronavirus*
cf: Conferre
CoV: Coronavirus
MERS-CoV: Middle-East Respiratory Syndrome betaCoV
N-RW: Not recent weaning
PPD: Posterior probability distribution
RW: Recent weaning
SARS-CoV: Severe Acute Respiratory Syndrome betaCoV

## References

[1] Mammal Diversity Database. 2019. www.mammaldiversity.org. American Society of Mammalogists. Accessed 20 Mar 2019.

[2] Ghanem SJ, Voigt CC, Brockmann HJ, Roper TJ, Naguib M, Mitani JC, Simmons LW (2012). Increasing awareness of ecosystem services provided by bats. Advances in the study of behavior.

[3] Kunz TH, Braun de Torrez E, Bauer D, Lobova T, Fleming TH (2011). Ecosystem services provided by bats. Ann N Y Acad Sci.

[4] Kalka MB, Smith AR, Kalko EKV (2008). Bats limit arthropods and herbivory in a tropical forest. Science.

[5] Williams-Guillén K, Perfecto I, Vandermeer J (2008). Bats limit insects in a neotropical agroforestry system. Science.

[6] Böhm SM, Wells K, Kalko EKV (2011). Top-down control of herbivory by birds and bats in the canopy of temperate broad-leaved oaks (*Quercus robur*). PLoS One.

[7] Maas B, Clough Y, Tscharntke T (2013). Bats and birds increase crop yield in tropical agroforestry landscapes. Ecol Lett.

[8] Federico P, Hallam TG, McCracken GF, Purucker ST, Grant WE, Correa-Sandoval AN (2008). Brazilian free-tailed bats as insect pest regulators in transgenic and conventional cotton crops. Ecol Appl.

[9] Cleveland CJ, Betke M, Federico P, Frank JD, Hallam TG, Horn J (2006). Economic value of the pest control service provided by Brazilian free-tailed bats in south-Central Texas. Front Ecol Environ.

[10] Boyles JG, Cryan PM, McCracken GF, Kunz TH (2011). Conservation. Economic importance of bats in agriculture. Science.

[11] Taylor PJ, Grass I, Alberts AJ, Joubert E, Tscharntke T (2018). Economic value of bat predation services--a review and new estimates from macadamia orchards. Ecosyst Serv.

[12] Ducummon SL (2000). Ecological and economic importance of bats.

[13] Muscarella R, Fleming TH (2007). The role of frugivorous bats in tropical forest succession. Biol Rev Camb Philos Soc.

[14] Fenolio DB, Graening GO, Collier BA, Stout JF (2006). Coprophagy in a cave-adapted salamander; the importance of bat guano examined through nutritional and stable isotope analyses. Proc Biol Sci.

[15] Gnaspini P, Trajano E, Wilkins H, Culver DC, Humphreys WF (2000). Guano communities in tropical caves. Ecosystems of the world subterranean ecosystems.

[16] Newman SH, Field H, Epstein J, de Jong C, Food and Agriculture Organization of the United Nations (2011). Investigating the role of bats in emerging zoonoses: balancing ecology, conservation and public health interest.

[17] Hayman DTS (2016). Bats as viral reservoirs. Annu Rev Virol.

[18] Calisher CH, Childs JE, Field HE, Holmes KV, Schountz T (2006). Bats: important reservoir hosts of emerging viruses. Clin Microbiol Rev.

[19] Woo PCY, Lau SKP, Li KSM, Poon RWS, Wong BHL, Tsoi H-W (2006). Molecular diversity of coronaviruses in bats. Virology.

[20] Vijaykrishna D, Smith GJD, Zhang JX, Peiris JSM, Chen H, Guan Y (2007). Evolutionary insights into the ecology of coronaviruses. J Virol.

[21] Drexler JF, Corman VM, Drosten C (2014). Ecology, evolution and classification of bat coronaviruses in the aftermath of SARS. Antivir Res.

[22] Anthony SJ, Johnson CK, Greig DJ, Kramer S, Che X, Wells H (2017). Global patterns in coronavirus diversity. Virus Evol.

[23] Li W, Shi Z, Yu M, Ren W, Smith C, Epstein JH (2005). Bats are natural reservoirs of SARS-like coronaviruses. Science.

[24] Anthony SJ, Gilardi K, Menachery VD, Goldstein T, Ssebide B, Mbabazi R, et al. Further evidence for bats as the evolutionary source of middle east respiratory syndrome coronavirus. MBio. 2017;8,e00373-17. 10.1128/mBio.00373-17 PMC5380844 28377531

[25] Ge X-Y, Li J-L, Yang X-L, Chmura AA, Zhu G, Epstein JH (2013). Isolation and characterization of a bat SARS-like coronavirus that uses the ACE2 receptor. Nature.

[26] Yuan J, Hon C-C, Li Y, Wang D, Xu G, Zhang H (2010). Intraspecies diversity of SARS-like coronaviruses in *Rhinolophus sinicus* and its implications for the origin of SARS coronaviruses in humans. J Gen Virol.

[27] Hu B, Ge X, Wang L-F, Shi Z (2015). Bat origin of human coronaviruses. Virol J.

[28] Ithete NL, Stoffberg S, Corman VM, Cottontail VM, Richards LR, Schoeman MC (2013). Close relative of human Middle East respiratory syndrome coronavirus in bat, South Africa. Emerg Infect Dis.

[29] Corman VM, Ithete NL, Richards LR, Corrie Schoeman M, Preiser W, Drosten C (2014). Rooting the phylogenetic tree of MERS-coronavirus by characterization of a conspecific virus from an African bat. J Virol.

[30] Lau SKP, Woo PCY, Li KSM, Huang Y, Tsoi H-W, Wong BHL (2005). Severe acute respiratory syndrome coronavirus-like virus in Chinese horseshoe bats. Proc Natl Acad Sci.

[31] Memish ZA, Mishra N, Olival KJ, Fagbo SF, Kapoor V, Epstein JH (2013). Middle East respiratory syndrome coronavirus in bats, Saudi Arabia. Emerg Infect Dis.

[32] World Health Organization (2004). Summary of probable SARS cases with onset of illness from 1 November 2002 to 31 July 2003.

[33] World Health Organization. Middle East respiratory syndrome coronavirus. https://www.who.int/emergencies/mers-cov/en/. Accessed 14 Oct 2018.

[34] Haagmans BL, Al Dhahiry SHS, Reusken CBEM, Raj VS, Galiano M, Myers R (2014). Middle East respiratory syndrome coronavirus in dromedary camels: an outbreak investigation. Lancet Infect Dis.

[35] Azhar EI, Hashem AM, El-Kafrawy SA, Sohrab SS, Aburizaiza AS, Farraj SA (2014). Detection of the Middle East respiratory syndrome coronavirus genome in an air sample originating from a camel barn owned by an infected patient. MBio.

[36] Azhar EI, El-Kafrawy SA, Farraj SA, Hassan AM, Al-Saeed MS, Hashem AM (2014). Evidence for camel-to-human transmission of MERS coronavirus. N Engl J Med.

[37] Chen L, Liu B, Yang J, Jin Q (2014). DBatVir: the database of bat-associated viruses. Database.

[38] Woo PCY, Lau SKP, Huang Y, Yuen K-Y (2009). Coronavirus diversity, phylogeny and interspecies jumping. Exp Biol Med.

[39] Pan Y, Tian X, Qin P, Wang B, Zhao P, Yang Y-L (2017). Discovery of a novel swine enteric alphacoronavirus (SeACoV) in southern China. Vet Microbiol.

[40] Corman VM, Baldwin HJ, Tateno AF, Zerbinati RM, Annan A, Owusu M (2015). Evidence for an ancestral association of human coronavirus 229E with bats. J Virol.

[41] Pfefferle S, Oppong S, Drexler JF, Gloza-Rausch F, Ipsen A, Seebens A (2009). Distant relatives of severe acute respiratory syndrome coronavirus and close relatives of human coronavirus 229E in bats, Ghana. Emerg Infect Dis.

[42] Yang X-L, Hu B, Wang B, Wang M-N, Zhang Q, Zhang W (2015). Isolation and characterization of a novel bat coronavirus closely related to the direct progenitor of SARS coronavirus. J Virol.

[43] Luo C-M, Wang N, Yang X-L, Liu H-Z, Zhang W, Li B (2018). Discovery of novel bat coronaviruses in south China that use the same receptor as MERS coronavirus. J Virol.

[44] Tao Y, Shi M, Chommanard C, Queen K, Zhang J, Markotter W (2017). Surveillance of bat coronaviruses in Kenya identifies relatives of human coronaviruses NL63 and 229E and their recombination history. J Virol.

[45] de Sales Lima FE, Campos FS, Kunert Filho HC, Batista HB d CR, Júnior PC, Cibulski SP (2013). Detection of *Alphacoronavirus* in velvety free-tailed bats (*Molossus molossus*) and Brazilian free-tailed bats (*Tadarida brasiliensis*) from urban area of southern Brazil. Virus Genes.

[46] Geller C, Varbanov M, Duval RE (2012). Human coronaviruses: insights into environmental resistance and its influence on the development of new antiseptic strategies. Viruses.

[47] Lau SKP, Li KSM, Huang Y, Shek C-T, Tse H, Wang M (2010). Ecoepidemiology and complete genome comparison of different strains of severe acute respiratory syndrome-related *Rhinolophus* bat coronavirus in China reveal bats as a reservoir for acute, self-limiting infection that allows recombination events. J Virol.

[48] Wacharapluesadee S, Duengkae P, Chaiyes A, Kaewpom T, Rodpan A, Yingsakmongkon S (2018). Longitudinal study of age-specific pattern of coronavirus infection in Lyle’s flying fox (*Pteropus lylei*) in Thailand. Virol J.

[49] Smith C (2017). Persistent or long-term coronavirus infection in Australian bats. Microbiol Aust.

[50] Baldwin HJ (2015). Epidemiology and ecology of virus and host: bats and coronaviruses in Ghana, West Africa.

[51] Drexler JF, Corman VM, Wegner T, Tateno AF, Zerbinati RM, Gloza-Rausch F (2011). Amplification of emerging viruses in a bat colony. Emerg Infect Dis.

[52] Wang M-N, Zhang W, Gao Y-T, Hu B, Ge X-Y, Yang X-L (2016). Longitudinal surveillance of SARS-like coronaviruses in bats by quantitative real-time PCR. Virol Sin.

[53] Jones KE, Patel NG, Levy MA, Storeygard A, Balk D, Gittleman JL (2008). Global trends in emerging infectious diseases. Nature.

[54] Greger M (2007). The human/animal interface: emergence and resurgence of zoonotic infectious diseases. Crit Rev Microbiol.

[55] Anthony SJ, Epstein JH, Murray KA, Navarrete-Macias I, Zambrana-Torrelio CM, Solovyov A (2013). A Strategy to estimate unknown viral diversity in mammals. mBio.

[56] Watanabe S, Masangkay JS, Nagata N, Morikawa S, Mizutani T, Fukushi S (2010). Bat coronaviruses and experimental infection of bats, the Philippines. Emerg Infect Dis.

[57] Quan P-L, Firth C, Street C, Henriquez JA, Petrosov A, Tashmukhamedova A (2010). Identification of a Severe Acute Respiratory Syndrome Coronavirus-like virus in a leaf-nosed bat in Nigeria. mBio.

[58] Kearse M, Moir R, Wilson A, Stones-Havas S, Cheung M, Sturrock S (2012). Geneious basic: an integrated and extendable desktop software platform for the organization and analysis of sequence data. Bioinformatics.

[59] Townzen JS, Brower AVZ, Judd DD (2008). Identification of mosquito bloodmeals using mitochondrial cytochrome oxidase subunit I and cytochrome b gene sequences. Med Vet Entomol.

[60] Benson DA, Cavanaugh M, Clark K, Karsch-Mizrachi I, Ostell J, Pruitt KD (2018). GenBank. Nucleic Acids Res.

[61] Ratnasingham S, Hebert PDN (2007). The barcode of life data system. Mol Ecol Notes.

[62] Jones KE, Bielby J, Cardillo M, Fritz SA, O’Dell J, Orme CDL (2009). PanTHERIA: a species-level database of life history, ecology, and geography of extant and recently extinct mammals. Ecology.

[63] Myhrvold NP, Baldridge E, Chan B, Sivam D, Freeman DL, Ernest SKM (2015). An amniote life-history database to perform comparative analyses with birds, mammals, and reptiles. Ecology.

[64] Luis AD, O’Shea TJ, Hayman DTS, Wood JLN, Cunningham AA, Gilbert AT (2015). Network analysis of host-virus communities in bats and rodents reveals determinants of cross-species transmission. Ecol Lett.

[65] Mühldorfer K, Speck S, Kurth A, Lesnik R, Freuling C, Müller T (2011). Diseases and causes of death in European bats: dynamics in disease susceptibility and infection rates. PLoS One.

[66] Hayman DTS, Suu-Ire R, Breed AC, McEachern JA, Wang L, Wood JLN (2008). Evidence of henipavirus infection in west African fruit bats. PLoS One.

[67] Amman BR, Carroll SA, Reed ZD, Sealy TK, Balinandi S, Swanepoel R (2012). Seasonal pulses of Marburg virus circulation in juvenile *Rousettus aegyptiacus* bats coincide with periods of increased risk of human infection. PLoS Pathog.

[68] Kolodny Oren, Weinberg Maya, Reshef Leah, Harten Lee, Hefetz Abraham, Gophna Uri, Feldman Marcus W., Yovel Yossi (2018). Coordinated change at the colony level in fruit bat fur microbiomes through time. Nature Ecology & Evolution.

[69] Willoughby AR, Phelps KL, Olival KJ, PREDICT Consortium (2017). A Comparative analysis of viral richness and viral sharing in cave-roosting bats. Diversity.

[70] Hoyt JR, Langwig KE, White JP, Kaarakka HM, Redell JA, Kurta A (2018). Cryptic connections illuminate pathogen transmission within community networks. Nature.

[71] van Schaik J, Kerth G (2017). Host social organization and mating system shape parasite transmission opportunities in three European bat species. Parasitol Res.

[72] Happold M, Happold D (2013). Mammals of Africa volume IV-hedgehogs, shrews and bats.

[73] Carpenter B, Gelman A, Hoffman MD, Lee D, Goodrich B, Betancourt M, et al. Stan: A probabilistic programming language. J Stat Softw. 2017;76(1) Available from: https://www.osti.gov/biblio/1430202. 10.18637/jss.v076.i01 PMC9788645 36568334

[74] R Core Team. R: A language and environment for statistical computing. R Foundation for Statistical Computing, Vienna, Austria. 2019. https://www.R-project.org/.

[75] Stan Development Team (2018). RStan: the R interface to Stan.

[76] Gelman A (2006). Prior distributions for variance parameters in hierarchical models (comment on article by Browne and Draper). Bayesian Anal.

[77] Polson NG, Scott JG (2012). On the half-Cauchy prior for a global scale parameter. Bayesian Anal.

[78] Gelman A, Rubin DB (1992). Inference from iterative simulation using multiple sequences. Stat Sci.

[79] Simmons NB, Wilson DE, Reeder DM (2005). Order Chiroptera. Mammal species of the world: a taxonomic and geographic reference.

[80] Vallo P, Benda P, Martínková N, Kauch P, Kalko EKV, Čeý J (2011). Morphologically uniform bats *Hipposideros aff. ruber* (*Hipposideridae*) exhibit high mitochondrial genetic diversity in southeastern Senegal. Acta Chiropt.

[81] Vallo P, Guillén-Servent A, Benda P, Pires DB, Koubek P (2008). Variation of mitochondrial DNA in the *Hipposideros caffer* complex (Chiroptera: Hipposideridae) and its taxonomic implications. Acta Chiropt.

[82] Happold M, Happold M, David H (2013). *Hipposideros ruber* Noack’s leaf-nosed bat. Mammals of Africa volume IV: bats and shrews.

[83] Happold M, Happold M, David H (2013). *Hipposideros cafer* Sundevall’s Roundleaf bat. Mammals of Africa volume IV: bats and shrews.

[84] Schweiger BR. Elucidating the species limits and range boundaries of the African yellow house bats, genus *Scotophilus*. Senior thesis. Lake Forest college, Illinois, US; 2017.

[85] Smith CS (2014). Australian bat coronaviruses.

[86] Gloza-Rausch F, Ipsen A, Seebens A, Göttsche M, Panning M, Drexler JF (2008). Detection and prevalence patterns of group I coronaviruses in bats, northern Germany. Emerg Infect Dis.

[87] Rihtarič D, Hostnik P, Steyer A, Grom J, Toplak I (2010). Identification of SARS-like coronaviruses in horseshoe bats (*Rhinolophus hipposideros*) in Slovenia. Arch Virol.

[88] Osborne C, Cryan PM, O’Shea TJ, Oko LM, Ndaluka C, Calisher CH (2011). Alphacoronaviruses in New World bats: prevalence, persistence, phylogeny, and potential for interaction with humans. PLoS One.

[89] Annan A, Baldwin HJ, Corman VM, Klose SM, Owusu M, Nkrumah EE (2013). Human betacoronavirus 2c EMC/2012-related viruses in bats, Ghana and Europe. Emerg Infect Dis.

[90] Peel AJ, Baker KS, Hayman DTS, Broder CC, Cunningham AA, Fooks AR (2018). Support for viral persistence in bats from age-specific serology and models of maternal immunity. Sci Rep.

[91] Plowright RK, Field HE, Smith C, Divljan A, Palmer C, Tabor G (2008). Reproduction and nutritional stress are risk factors for Hendra virus infection in little red flying foxes (*Pteropus scapulatus*). Proc Biol Sci.

[92] Epstein JH, Baker ML, Zambrana-Torrelio C, Middleton D, Barr JA, Dubovi E (2013). Duration of maternal antibodies against canine distemper virus and Hendra virus in pteropid bats. PLoS One.

[93] Peel AJ, Baker KS, Crameri G, Barr JA, Hayman DTS, Wright E (2012). Henipavirus neutralising antibodies in an isolated island population of African fruit bats. PLoS One.

[94] Breed AC, Breed MF, Meers J, Field HE (2011). Evidence of endemic Hendra virus infection in flying-foxes (*Pteropus conspicillatus*)—implications for disease risk management. PLoS One.

[95] Jeong J, Smith CS, Peel AJ, Plowright RK, Kerlin DH, McBroom J (2017). Persistent infections support maintenance of a coronavirus in a population of Australian bats (*Myotis macropus*). Epidemiol Infect.

[96] Peel AJ, Wood JLN, Baker KS, Breed AC, Carvalho AD, Fernández-Loras A (2017). How does africa’s most hunted bat vary across the continent? Population traits of the straw-coloured fruit bat (*Eidolon helvum*) and its interactions with humans. Acta Chiropt.

[97] Dietrich M, Wilkinson DA, Benlali A, Lagadec E, Ramasindrazana B, Dellagi K (2015). Leptospira and paramyxovirus infection dynamics in a bat maternity enlightens pathogen maintenance in wildlife. Environ Microbiol.

[98] Tong S, Conrardy C, Ruone S, Kuzmin IV, Guo X, Tao Y (2009). Detection of novel SARS-like and other coronaviruses in bats from Kenya. Emerg Infect Dis.

[99] DeFrees SL, Wilson DE (1988). Eidolon helvum. Mamm Species.

[100] Dengis CA (1996). Taphozous mauritianus. Mamm Species.

[101] Dunlop J (1997). Coleura afra. Mamm Species.

[102] Independent Scientific Group on Cattle TB (2007). Bovine TB: the scientific evidence.

[103] Comte S, Umhang G, Raton V, Raoul F, Giraudoux P, Combes B (2017). *Echinococcus multilocularis* management by fox culling: an inappropriate paradigm. Prev Vet Med.

[104] Morters MK, Restif O, Hampson K, Cleaveland S, Wood JLN, Conlan AJK (2013). Evidence-based control of canine rabies: a critical review of population density reduction. J Anim Ecol.

[105] Streicker DG, Recuenco S, Valderrama W, Gomez Benavides J, Vargas I, Pacheco V (2012). Ecological and anthropogenic drivers of rabies exposure in vampire bats: implications for transmission and control. Proc Biol Sci.

[106] Blackwood JC, Streicker DG, Altizer S, Rohani P (2013). Resolving the roles of immunity, pathogenesis, and immigration for rabies persistence in vampire bats. Proc Natl Acad Sci.

[107] Fornes A, Lord RD, Kuns ML, Larghi OP, Fuenzalida E, Lazara L (1974). Control of bovine rabies through vampire bat control. J Wildl Dis.

[108] Amman BR, Nyakarahuka L, McElroy AK, Dodd KA, Sealy TK, Schuh AJ (2014). Marburgvirus resurgence in Kitaka mine bat population after extermination attempts, Uganda. Emerg Infect Dis.

[109] Towner JS, Amman BR, Sealy TK, Carroll SAR, Comer JA, Kemp A (2009). Isolation of genetically diverse Marburg viruses from Egyptian fruit bats. PLoS Pathog.

[110] Maganga GD, Bourgarel M, Ella GE, Drexler JF, Gonzalez J-P, Drosten C (2011). Is Marburg virus enzootic in Gabon?. J Infect Dis.

[111] López-Roig M, Serra-Cobo J (2014). Impact of human disturbance, density, and environmental conditions on the survival probabilities of pipistrelle bat (*Pipistrellus pipistrellus*). Popul Ecol.

[112] Field HE (2009). Bats and emerging zoonoses: henipaviruses and SARS. Zoonoses Public Health.

[113] Kirkpatrick JF, Turner JW (1991). Compensatory reproduction in feral horses. J Wildl Manag.

[114] Langvatn R, Loison A (1999). Consequences of harvesting on age structure, sex ratio and population dynamics of red deer *Cervus elaphus* in Central Norway. Wildlife Biol.

[115] Beasley JC, Olson ZH, Beatty WS, Dharmarajan G, Rhodes OE (2013). Effects of culling on mesopredator population dynamics. PLoS One.

[116] Bonesi L, Harrington LA, Maran T, Sidorovich VE, Macdonald DW (2006). Demography of three populations of American mink mustela vison in Europe. Mamm Rev.

[117] Cowan PE (1993). Effects of intensive trapping on breeding and age structure of brushtail possums, *Trichosurus vulpecula*, on Kapiti Island, New Zealand. N Z J Zool.

[118] Lindahl JF, Grace D (2015). The consequences of human actions on risks for infectious diseases: a review. Infect Ecol Epidemiol.

[119] Morse SS, Price-Smith AT (2001). Factors in the emergence of infectious diseases. Plagues and politics: infectious disease and international policy.

[120] Murray KA, Daszak P (2013). Human ecology in pathogenic landscapes: two hypotheses on how land use change drives viral emergence. Curr Opin Virol.

[121] Ceballos G, Ehrlich PR, Barnosky AD, García A, Pringle RM, Palmer TM (2015). Accelerated modern human–induced species losses: entering the sixth mass extinction. Sci Adv.

[122] Voigt CC, Kingston T, Voigt CC, Kingston T (2016). Bats in the Anthropocene. Bats in the Anthropocene: conservation of bats in a changing world.

[123] Buttke DE, Decker DJ, Wild MA (2015). The role of one health in wildlife conservation: a challenge and opportunity. J Wildl Dis.

[124] Terraube J, Fernández-Llamazares Á, Cabeza M (2017). The role of protected areas in supporting human health: a call to broaden the assessment of conservation outcomes. Curr Opin Environ Sustain.

