# Supplementary material for: Reproduction of East-African bats may guide risk mitigation for coronavirus spillover
Source: One Health Outlook. 2020 Feb 7;2:2. doi: 10.1186/s42522-019-0008-8 (PMC7149079; doi:10.1186/s42522-019-0008-8)
Supplement: Supplementary file 1 — Additional file 1: Summary of the inferred start date of the birth pulse, the end of the lactation period, and the start date of the mating period per microchiropteran species. [file 42522_2019_8_MOESM1_ESM.docx]

**Additional file 1**

Summary of the inferred start date of the birth pulse, the end of the lactation period, and the start date of the mating period per microchiropteran species.

***Chaerephon pumilus***

To adequately infer the reproductive season of this species, we also searched for the scientific literature on the historical synonyms of this species: *Chaerephon leucogaster*, *Chaerephon pumila*, *Chaerephon pusillus*, *Tadarida leucogaster*, *Tadarida pumila*, *Tadarida pusilla*, *Tadarida cristatus, Tadarida elphicki, Tadarida faini, Tadarida frater, Tadarida gambianus, Tadarida hindei, Tadarida langi, Tadarida limbata, Tadarida naivashae, Tadarida nigri, Tadarida pusillus, Tadarida websteri* [1,2]**.**

*Chaerephon pumilus* samples were collected in Tanzania (n=123), Rwanda (n=3), and in Uganda (n=1) in the months of February, March, April, August, and November. No lactating female was captured. Three females were captured while pregnant in April and November. The two pregnant females captured in April were sampled with one year of difference. Non-adult individuals were captured in April, August, and November.

There are several reports in this species related to the presence of pregnant and/or lactating females and size of testes in males across different latitudinal degrees and there are clear differences in the reproductive cycle at different latitudes.

At some locations, females could show postpartum estrus with a pregnancy while still nursing their offspring. Here we will follow the calendar proposed by [3] interpreting the data from [4] and [5]. Both of these authors followed a colony of this species for one year in Uganda. These authors propose that it is likely that each female has 3 births per year and the last birth is not followed by postpartum estrus but by a posterior reproductive inactivity [6]. The percentage of heavily pregnant females fluctuates from 9 to 71%, with peaks occurring in October (71%), February (53%) and June-July (87%, 70%). These peaks are followed by an abrupt drop when most births occur: November-December, March-April and July-August. In these peaks there are groups of females in reproductive synchronization. Data suggest that females give birth at intervals of 4 months and, therefore, *C. pumilus* avoids the co-occurrence of pregnancy and lactation in Kenya and Uganda [4,7]. Biologists have supported that this species is seasonally polyoestrous, and able to conceive as many as 3 times per breeding season in South Africa [8]. Consequently, we assigned March, July, and November 1^st^  as the start dates of the birth pulses. Furthermore, because the birth pulses last about two months each (see above) and this species experiences a lactation period of 3 weeks[4,5], we inferred that the first weaned pups aroused in March, July, and November 22nd, while the lactation period was over after 3 weeks, ending on April 30^th^, August 31^st^, and December 31^st^. Therefore, the “Recent weaning” period lasts until February, June, and October 21^st^.

***Coleura afra***

To adequately infer the reproductive season of this species, we also searched for scientific literature on the historical synonyms of this species: *Emballonura afra*, *C. gallarum*, *C. kummeri*, *C. nilosa [1]***.**

*Coleura afra* samples were collected in Tanzania (n=7) during June and October. No pregnant female, lactating female, or female carrying a pup was captured. No Non-adult individuals were captured neither.

In Kenya, it was found that this species had a bimodal cycle [9]. Two birth periods were also described in Ghana [10], while a single parturition period at a northern latitude in Sudan [11]. This species was classified as monoestrous [12]. There seems to be no data supporting polyestry in this species.

Following the data from Kenya, the testes of males increase in size during the rainy seasons and peak about the end of the rainy season, when copulation occurs [9]. If this is correct, then copulation occurs in July and in December and the birth pulse occur 16 weeks later: in March - April and November - December, while lactation after the first birth pulse matches the long rainy seasons: April - July and November - February, respectively. The presence of young *C. afra* since November in Kenya was reported [13] , while a pregnant female was reported in Amboni caves in December (Tanzania, one of the actual sampling locations) [14]. Postpartum estrus was suggested as feasible [9] that but other biologists said that this adaptation is not present in this species [15].

Because of the difference in latitude between Kenya and Tanzania, and the reported pregnant female in Tanzania during December we inferred that individuals of this species are somewhat delayed with respect to Kenya. Consequently, we assigned March and November 15^th^ as the start dates of the birth pulses. Furthermore, because the birth pulses last about two months each (see above) and this species presents a lactation period of 4 weeks [9] we inferred that the first weaned pups aroused in December and April 15^th^. Therefore, the “Recent weaning” period lasts until February, June, and October 21^st^.

***Eidolon helvum***

To adequately infer the reproductive season of this species, we also searched for scientific literature on the historical synonyms of this species: *Vespertilio vampirus ssp. Helvus*.

*Eidolon helvum* samples were collected in Tanzania (n=265) in the months of January, April, June, August, and September. A pregnant female was sampled in late January, lactating females were found in late January and late April, and a female with the pup attached was captured in late January. Non-adult individuals were captured in January (n=1), April (n=23), June (n=29), August (n=21), and September (n=1).

With respect to previous literature, very early and early pregnancies were reported during August in Dar es Salaam, Tanzania, as well as sexually immature individuals between 6 - 12 months old and between 18 - 24 months old [16,17]. Individuals of these ages are present in an colony 6 to 12 months after the birth pulse [18]. Data from Morogoro, Tanzania, was consistent with the findings for Dar es Salaam (the distance between the 2 cities is ~ 180 km) [18] and a personal communication mentions suckling neonates attached to flying females and some juveniles flying independently during January in Morogoro [16]. Because *E. helvum* breeds once a year and shows seasonally synchronized birth pulses (e.g., [19,20]), these data strongly suggest a birth pulse in the second part of December and January-February. Therefore, we followed [17] and assigned December 15^th^ as the start date of the birth pulse in this country. The annual birth pulse lasts about 2.5 - 3 months [19,20]; therefore, we infer that the last lactating females aroused in March 15th. Our inferred cycle resembles the one proposed by [21].

The time-to-weaning was defined based on PanTHERIA and Amniote databases [22,23] which report ~60 days; therefore, the first pups weaned occurred in mid-February, and the last ones by mid-May. From here, the “Recent weaning” period lasts until the end of the first week of June.

***Hipposideros sp.***

To adequately infer the reproductive season of these species, we also searched for scientific literature on the historical synonyms of the two common hipposideros species present in the area where these bats were captured for sampling: *H. caffer* and *H. ruber*. These are *Rhinolophus caffer*, *H. angolensis*, *H. aurantiaca*, *H. bicornis*, *H. braima, H. gracilis*, and *H. nanus,* for *H. cafer;* and *H. centralis*, *H. niapu*, and *H. ruber* ssp. *Guineensis* for *H. ruber*. Today, *Hipposideros caffer/ruber* are recognized as a species complex [24,25].

Caution must be taken with these species in this Genera. According to the International Union for the Conservation of Nature red list of threatened species, this group is complex and requires taxonomic revision. The Paleotropical family of the leaf-nosed bats, Hipposideridae has traditionally had a difficult taxonomy due to the extreme morphological similarity of many of its members, whilst they are genetically variable [24–26].

*Hipposideros sp.* samples were collected in Tanzania (n=15), Uganda (n=3), and Rwanda (n=4) during May, August, and September. No females were captured pregnant, lactating or with young. No non-adults were captured.

The reproductive biology of this genus (both species of concern) follow a restricted seasonal monoestrous cycle [1]. In Tanzania retarded development seems to occur and claims a boreal and a austral cycle is present in *H. caffer* but the equatorial populations can follow either cycle [27,28]. However, it has been suggested that different cycles could be different species [29], although no further argumentation was provided; however, *H. caffer* in Tanzania was found to be genetically distinct from *H. caffer* sampled in Uganda [24].

The boreal cycle is supported by the data presented in [30–32]. Here we follow what has been described as the austral cycle because of the latitudes where these bats lived, and it has proposed that this cycle is observed in Tanzania [33].

Data from Gabon (~ latitude 0) described that in the austral cycle pregnant females are found between July-August and that after 80 - 100 days, the birth pulse occurs in October-November [28]. Lactation was observed until the end of January. Afterwards these bats show reproductive quiescence until June. The data presented by these authors is the more convincing southern cycle of this species. Special attention is required with this reference as the figure showing the Boreal and Austral cycle has the labels reversed compared with the actual description in the main text. Data from South Africa is consistent with the previously described cycle reporting that during August females were pregnant [34], although the latitude is much southern relative to Tanzania.

In the case of *H. ruber*, [21] cites [35] and points out that in Tanzania (~ 7S), females are in gestation during November and do not present reproductive activity in May. Consistently, it was claimed that at this latitude the copulation, ovulation, and fertilization occur in June - July, parturition is mainly in December and that lactation occurs between December and February [36]. In Equatorial Guinea, ~1.65 N very similar to Uganda in latitude but in West Africa, *H. caffer* was found with large embryos in October and lactating in November [37]; however, it is suspected that this author is actually describing *H. ruber [1]*. In Sao Tome Island ~0N the timing of pregnancy period for *H. ruber* on the island was September–November and late lactation–post lactation in January 2010 [38]. These descriptions are consistent with each other and they resemble the cycle described for *H. caffer*.

All references are consistent with a long and delayed pregnancy between June and October, with copulation around June, births in November, and lactation from November to January. Therefore, we assigned October 21^st^ as the start date of the birth pulses. Although this could be misplaced by a couple of weeks, it would not make a difference with respect to the bats captured, as they were sampled in months distant from the birth pulse and the lactation periods. Furthermore, because the birth pulses last about two months (see above) and that lactation occurred for 3-4 months [28,39], we inferred that the first weaned pups were present at the end of December, and the “Recent weaning” period lasts until February 28^th^.

***Lissonycteris*** ***angolensis (Mynonycteris angolensis* and *Rousettus angolensis)***

Considered the single representant of its genus. But it has been taxonomically modified several times, and therefore, we also searched for scientific literature on the historical synonyms of this species: *Lissonycteris goliath*, *L. petraea L. ruwenzorii L. smithii,*  and *Rousettus angolensis* [40]. Furthermore, we searched for the genus *Myonycteris* followed by the species names [1].

In the dataset there are 49 individuals, 46 of them captured in Rwanda and three in Uganda. The individuals captured in Rwanda were sampled in April (n=16) and May (n=30). Furthermore, a single individual was captured in February, July, and November in Uganda. In Rwanda, three females were captured when lactating during April (n=2) and May (n=1).

The information on the reproductive cycle of this species is scattered. In Nigeria, six pregnant females and one lactating female were reported during July [41]. In Liberia, 71% (35/49) of females were found pregnant during August. These females were ready to give birth in September [42]. Two pregnant females were found during December in this country [43]. A personal communication in [44] reported a pregnant female and 2 females that gave birth in November, and finally, pregnant females were reported in October and December [45], suggesting the species produce young in early December.

In Bioko Island, East Africa, northern than the study area (~3 N), 29 of 32 females were found pregnant and the remaining three were reproductively inactive in February [46]. This author also reported five pregnant females of the five captured in March, nine pregnant females of 16 captured in October, two pregnant females of three captured in November and one lactating female. Finally, two out of four captured females were lactating in December. This suggests polyoestry. In Congo, near parturient females have been recorded from May to September and in November, and lactation in January, February, and June [47]. In Ethiopia, females at mid-stages of pregnancy at the end of April and they were observed at the beginning of May also, but less than half of females were pregnant [42].

The reproductive chronology is uncertain, probably with polyestry at least in some localities [1]. For these reasons, the seasons will be assigned by modeled imputation.

***Mops condylurus***

To adequately infer the reproductive season of this species, we also searched for scientific literature on the historical synonyms of this species: *Mops angolensis*, *Mops fulva*, *Mops occidentalis*, *Mops orientis*, *Mops osborni*, *Mops wonderi*, *Nyctinomus condylurus*, and *Tadarida condylura* [1]*.*

*Mops condylurus* samples were collected in Tanzania (n=42) during April, August, and November. One female was captured while pregnant at the end of April. Three non-adult individuals were captured in April.

A study conducted at 0’43 N in Uganda for 2 years showed that this species at this latitude has a bimodal breeding rhythm closely correlated with the rainfall pattern [4]. This author found pregnancies in May-July and December-January, which is concordant with the pregnant female at the end of April (27^th^). Furthermore, a bimodal pattern was also found in Kenya (2.18°S) with the parturient seasons in March-April and November [7] matching our pregnant female. Data from Malawi and expanded the data from Kenya via personal communication with the previous authors confirmed the bimodal pattern with parturitions; however, there is a postpartum estrus after November’s birth pulse. Affter the second birth pulse in April there is not a postpartum estrus. Instead, a reproductive quiescence period begins, which would include the month of August [3].

Following Malawi and Kenya data, and also the monthly average pattern of precipitation of the last 20 years in the sampling locations (TerraClimate), we assigned March and November 1^st^  as the start dates of the birth pulses. Furthermore, because the birth pulses last about two months and this species experiences a lactation period of 6-7 weeks [48] we inferred that the first weaned pups occurred in December and April 15^th^. Therefore, the “Recent weaning” period lasts until March and July 15^th^.

***Neoromicia nana***

To adequately infer the reproductive season of this species, we also searched for scientific literature on the historical synonyms of this species: *Pipistrellus nanus (Peters, 1852), Vespertilio nanus (Peters, 1852),* and *Neoromicia nanus [1]***.**

*Neoromicia nana* samples were collected in Tanzania (n=1) and Uganda (n=8) during April, July, and November. No female was captured while pregnant, lactating or carrying a pup. A single non-adult individual was captured in November.

A pregnant female and young of various ages from January to April and from June to August in Gabon [49]. In Tanzania, 1 of 49 females was pregnant, and no juveniles or scrotal males were found between July and August [50]. Also, in Tanzania, two males, both with abdominal testes and three males with scrotal testes, were captured in August and females collected in July were nulliparous, except for one pregnant female [51]. In Uganda, two post parous females with large nipples and 3 males with large scrotal testes in May [52]. A specimen netted in October was also an adult female with large nipples but not pregnant. Furthermore, these authors report an adult pregnant female in June. In Rwanda breastfeeding newborn infants were found in February and November; and embryos, infants, and young of various ages were found at the end of January, February and April [53]. Finally, in Kenya, no external evidence of testicular activity was observed from October to March, but the spermatogenic cycle started in this last month and lasted to September based on scrotal testes. It was assumed that the season of copulation extends from May through late August. The onset of gestation would occur during late August or early September and the parturient season corresponds to November as pregnant females were noted only immediately prior to the November rains. Lactation was observed in November - January [7,54]. Although aseasonal breeding has been suggested for Rwanda, and Gabon and Congo [39,53], data would not allow these authors to determine whether the chronology is aseasonal monoestry with highly unsynchronized parturition or aseasonal polyoestry [55]. However, none of these references included the most complete study of reproduction in this species conducted in Kenya [56] that reported parturition in October and November as previously mentioned and projects the reproductive cycle of this species to be similar to *P. hesperus* (North America) which occurs in temperate climates.

Due to the geographic closeness of Rwanda, Tanzania, Kenya and Uganda and their similar latitudes, we followed the evidence in all countries. It seems clear that there is a birth pulse in October-November after pregnancy between July and October. Therefore, we assigned October 15^th^ as the start date of the birth pulse. The individuals sampled in November were inferred to be in “Recent weaning”. We followed the suggestion that the species is monoestrous. The other sampled *N. nana* in this study were far from the end of lactation period assigned and from the end of the inferred “Recent weaning” period.

***Nycteris thebaica***

This species does not have historical synonyms.

*Nycteris cf. thebaica s*amples were collected in Rwanda (n=5) during April and May. No females were captured while pregnant, lactating or carrying pups. No non-adult individuals were sampled.

The literature about the reproduction of this species is limited. At 8S in Congo, pregnant females were reported in August and absence of reproduction in February-March, June-July and October-December [33]. However, this could be related to the sample size. This author also reported females with juveniles captured in November and December in Tanzania at 8S, a female with a pup in January at 6S, and females lactating large juveniles at the end of October at 2.25S. Furthermore, this author also reported a pregnant female and a female lactating in October in Rwanda at 3S, and a newborn and a pregnant female close to term in the first half of July at 2S in this country. Finally, this author also found a near term female and females bearing juveniles in West Africa at 1S in February.

It was claimed that reproduction is polyestrous and continuous with lactating females becoming pregnant [33]. Moreover, it was mentioned the possibility of two litters in rapid succession because in Rwanda, mating would occur in April, birth in July, while in Tanzania and Kenya, copulation occurs in October-November, births in January-February and lactation ended in April [33]. However, other biologists claim that these conclusions are misleading and invalid [57]. In Tanzania at 7S and[7] in Kenya at 2S a restricted seasonal monoestry with births in November was found [7,35] which is supported by [57].

Considering the description in Kenya, we assigned November 1^st^  as the start date of the birth pulse. Furthermore, because lactation ins this species lasts for 2 months[58], the “Recent weaning” period would start in January 1^st^. From here birth pulses could extend for anywhere between one to two months and the sampled individuals would still be inferred as in the “Not recent weaning” period.

***Pipistrellus hesperidus***

To adequately infer the reproductive season of this species, we also searched for scientific literature on the historical synonyms of this species: *Vespertillio hesperida, Pipistrellus kuhlii, Pipistrellus broomi, P. fuscatus, P. subtilis.* If there is a subspecies, *P. hesperidus fuscatus* is the one located in East Africa[1]. There is a *P. hesperus* which is an American species. Moreover, *P. kuhlii* now is considered a species in Europe and very North Africa (shore of the Mediterranean Sea) [1]**.**

*Pipistrellus cf. hesperidus s*amples were collected in Uganda (n=7) during February. No females were captured while pregnant, lactating or carrying pups. No non-adult individuals were sampled.

There is not enough information in the current literature to establish reproductive life history at the corresponding latitude.

***Rousettus aegyptiacus***

To adequately infer the reproductive season of this species, we also checked the literature for the following species synonyms: *arabicus, egyptiacus, geoffroyi, hottentotus, leachii, occidentalis, princeps, sjostedti, thomensis, tomensis, unicolor* and *Pteropus aegyptiacus*.

*Rousettus aegyptiacus* samples were collected in Tanzania (n=4) in April and in Rwanda (n=39) during May and September. Three pregnant females were sampled in mid-May, nine lactating females were found in mid-May (n=3) and late September (n=6). Six of these lactating females were carrying their pups. Non-adult individuals were captured in May (n=4).

With respect to previous literature, year-round or seasonal breeding has been observed depending on the latitude (monoestry or polyoestry and both seasonal and non-seasonal chronologies are found). In Uganda at latitude 0 breeding is continuous but with 2 peaks of pregnancies: December - February and July – October. The corresponding birth pulses occur in March and in September - October[59–61]. May and November are described as peak periods of breeding[61] and the former month has been described as a month when females can be lactating and can be pregnant[60]. Postpartum estrus is supported by data[59,62,63]. Pregnant females have been observed during August in this country[64]. In Tanzania, pregnant females were observed in August[51]. Finally, in Kenya a birth season in July – August was claimed[39]; however, no supporting data was presented.

Considering the latitude similarity between Uganda and Rwanda, as well as the pregnant females in May and the lactating females carrying their young in September, we inferred that March 1^st^ and September 1^st^ as the starting date of the birth pulse. The annual birth pulse lasts about 3 months [59–61], therefore, the last lactating females aroused in June and December 1^st^. The time-to-weaning was defined in 2 months [59,63,65] which report ~70 days. Consequently, the first pups weaned occurred in May and November, and the last ones by August and February. From here, the “Recent weaning” period lasts until September and March.

***Rhinolophus clivosus***

To adequately infer the reproductive season of this species, we also searched for scientific literature on the historical synonyms of this species: *Rhinolophus acroti , R. andersoni, R. augur, R. brachygnatus, R. keniensis, R. schwarzi, R zambesiensis and R. zuluensis [1].* Variability in several features suggests there is more than one species. Subspecies *keniensis* is previously recognized in Kenya, Uganda and northern Tanzania. The subspecies *augur* occurs in Tanzania; however, this species has not been recorded in Tanzania and that previous findings corresponded to *R. deckinii* and *R. fumigatus* according to [1]*.* Further taxonomic research is needed into *Rhinolophus clivosus* as this may represent a complex of several species.

*Rhinolophus cf. clivosus s*amples were collected in Uganda (n=39) and Rwanda (n=22). during May and September. No females were captured while pregnant, lactating or carrying pups, and nine non-adult individuals were sampled.

With respect to the reproductive cycle, restricted seasonal monoestry has been reported in Zimbabwe and South Africa. In Zimbabwe births occur during the dry season (June-July, winter). In South Africa copulation takes place in April with posterior sperm storage until August (late winter) and parturition in summer (November December). However, at a northern latitude in South Africa, the cycle is totally different. For this reason, the possibility of there being 2 different species has been raised [1]. In Tanzania, 2 females with large teats, several females with non-lactating teats, and 3 of 6 males had scrotal testes during August [51]. These authors also report a female with an embryo in August and a non-reproductive female. Similarly, some males had scrotal testes during July-September.

Inferring the reproductive season based on previous research is not adequate. Better data supporting a reproductive cycle were obtained from remarkably higher latitudes than the sites where the bats in our dataset were captured. There is not enough information to establish reproductive life history at the corresponding latitude.

***Taphozous mauritianus***

To adequately infer the reproductive season of this species, we also searched for scientific literature on the historical synonyms of this species: *cinerascens, dobsoni,* and *leucopterus.*

*Taphozous mauritianus s*amples were collected in Tanzania (n=11) during the end of November. No females were captured while pregnant, two females were lactating and both of them were carrying a juvenile. Moreover, a single juvenile and one non-adult individual were sampled.

Our findings are consistent with [53]. This author claims that in northern Zaire, Tanzania, and Kenya, births occurred in April-May, and in October-November. Further, the parturient season was established to occur in April, May, and November [7]. Other similar results have been reported in other latitudes. For example, births occur at least in November-December and in March-April at 9S [6], births in November were reported in Tanzania [66], a female with a pup in November at 15S [67], and in October at 30S [68,69]. Perhaps at higher latitudes the cycle is displaced by about one month.

Considering the descriptions previously provided and the presence of females with pups and the non-adult sampled, we assigned November 7^th^ as the start date of the birth pulse. Although the lactation length is unknown, we believe that the sampled individuals were too close to the start of the birth pulse, and that they were not in the “Recent weaning period” but in “Not recent weaning”.

***Triaenops persicus***

This species does not have historical synonyms; however, this species is currently named *Triaenops* *afer* when within Africa [1,70].

Samples from this species were collected in Tanzania (n=46) during June. No females were captured while pregnant, lactating or carrying pups, and three non-adult individuals were sampled.

Information about the biology of this species is scarce, but there are 3 records of pregnancies in Tanzania, all of them in December [35,70,71]. Births in this species were described to occur in December and January while pregnancy was observed starting in October [35]. Fluctuations in spermatogenic activity are concurrent with the size of testes [72] and testicular enlargement in *T. Persicus* is at its peak during June and July [35].

Following a single peak in testicular enlargement, we can infer that this species experiences one parturition per year (similar to other hipposiderids) that starts on December 1^st^. Lactation length could be as in other hipposiderids or even longer, but the sampled members of this species would still be in “No recent weaning”.

**References**

1. Happold M, Happold D, editors. 2013 *Mammals of Africa Volume IV-Hedgehogs, Shrews and Bats*. London, UK: Bloomsbury Publishing.

2. Mickleburgh S, Hutson AM, Racey PA, Ravino J, Bergmans W, Cotterill F, Gerlach J. 2014 *Chaerephon pumilus*. *The IUCN Red List of Threatened Species*. See http://www.iucnredlist.org/details/4317/0 (accessed on 28 September 2017).

3. Happold DCD, Happold M. 1989 Reproduction of Angola free-tailed bats (*Tadarida condylura*) and little free-tailed bats (*Tadarida pumila*) in Malawi (Central Africa) and elsewhere in Africa. *J. Reprod. Fertil.* **85**, 133–149.

4. Mutere FA. 1973 Reproduction in two species of equatorial free-tailed bats (Molossidae). *Afr. J. Ecol.* **11**, 271–280.

5. Marshall AJ, Corbet PS. 1959 The breeding biology of equatorial vertebrates: reproduction of the bat *Chaerephon hindei* Thomas at latitude 0 26′ N. *Proc. Zool. Soc. Lond.* ***132***, 607–616.

6. Happold DCD, Happold M. 1990 Reproductive strategies of bats in Africa. *J. Zool.* **222**, 557–583.

7. O’Shea TJ, Vaughan TA. 1980 Ecological observations on an East African bat community. *Mammalia* **44**, 485–496.

8. Van der Merwe M, Rautenbach IL, Van der Colf WJ. 1986 Reproduction in females of the little free-tailed bat, *Tadarida* (*Chaerephon*) *pumila*, in the eastern Transvaal, South Africa. *J. Reprod. Fertil.* **77**, 355–364.

9. McWilliam AN. 1987 The reproductive and social biology of *Coleura afra* in a seasonal environment. In *Recent Advances in the Study of Bats* (eds MB Fenton, P Racey, and MV Rayner,), pp 324-350 Cambridge, UK: Cambridge University Press.

10. McWilliam AN. 1976 The biology of *Tadarida (Chaerephon) pumila* (Cretzschmar) and partitioning of food resources among insectivorous bats in northern Ghana. B. Sc. Hons thesis. University of Aberdeen, Aberdeen, Scotland.

11. Kock D. 1969 Die Fledermaus-Fauna des Sudan. *Abh. Senckenberg. Naturf. Ges*. **521,** 1-23.

12. Anciaux de Faveaux M. 1973 Essai de synthese sur la réproduction de chiropteres d’Afrique (Région Faunistique Ethiopienne). *Period. Biol.* **75**, 195–199.

13. Makori B. 2015 Survey and conservation of cave-dwelling bats in coastal Kenya. Karatina University, School of Natural Resources and Environmental Studies.

14. Matthews LH. 1942 Notes on the genitalia and reproduction of some African rats. *Proc. Zool. Soc. Lond.* B, **111**, 289–342.

15. Happold M, Others. 2013 *Coleura afra* African sheath-tailed bat. In *Mammals of Africa Volume IV: Hedgehogs, Shrews*, *and Bats* (eds M Happold, D Happold), pp. 422-424. London, UK: Bloomsbury Publishing.

16. Peel AJ. 2012 The epidemiology of Lagos bat virus and henipaviruses in straw-coloured fruit bats (*Eidolon helvum),* using population genetics to infer population connectivity. PhD Dissertation. Wolfson College, Cambridge, UK.

17. Peel AJ *et al.* 2017 How does Africa’s most hunted bat vary across the continent? Population traits of the straw-coloured fruit bat (*Eidolon helvum*) and its interactions with humans. *Acta Chiropt.* **19**, 77–92.

18. Peel AJ *et al.* 2016 Bat trait, genetic and pathogen data from large-scale investigations of African fruit bats, *Eidolon helvum*. *Sci Data* **3**, 160049.

19. Mutere FA. 1967 The breeding biology of equatorial vertebrates: reproduction in the fruit bat, *Eidolon helvum*, at latitude 0 20′ N. *J. Zool.* **153**, 153–161.

20. Funmilayo O. 1979 Ecology of the straw-coloured fruit bat in Nigeria. *Revue de Zoologie Africaine* **93**, 589–600.

21. Bernard RT, Cumming GS. 1997 African bats: evolution of reproductive patterns and delays. *Q. Rev. Biol.* **72**, 253–274.

22. Myhrvold NP, Baldridge E, Chan B, Sivam D, Freeman DL, Ernest SKM. 2015 An amniote life-history database to perform comparative analyses with birds, mammals, and reptiles. *Ecology* **96**, 3109–3109.

23. Jones KE *et al.* 2009 PanTHERIA: a species-level database of life history, ecology, and geography of extant and recently extinct mammals. *Ecology* **90**, 2648–2648.

24. Vallo P, Guillén-Servent A, Benda P, Pires DB, Koubek P. 2008 Variation of mitochondrial DNA in the *Hipposideros caffer* complex (Chiroptera: Hipposideridae) and its taxonomic implications. *Acta Chiropt.* **10**, 193–206.

25. Vallo P, Benda P, Martínková N, Kauch P, Kalko EKV, Čeý J, Koubek P. 2011 Morphologically uniform bats *Hipposideros* *aff. ruber* (Hipposideridae) exhibit high mitochondrial genetic diversity in southeastern Senegal. *Acta Chiropt.* **13**, 79–88.

26. Simmons NB. 2005 Order Chiroptera. In *Mammal Species of the World: A taxonomic and Geographic Reference* (eds DE Wilson and DM Reeder), pp. 312–529. Baltimore, Maryland, US: Johns Hopkins University Press.

27. Wright GS. 2009 *Hipposideros caffer* (Chiroptera: Hipposideridae). *Mammalian Species*, **845**, 1-9.

28. Brosset A, Saint Girons H. 1980 Cycles de reproduction des microchiroptères troglophiles du nord-est du Gabon. *Mammalia* **44**, 225–232.

29. Happold M. 2013 *Hipposideros caffer* Sundevall’s roundleaf bat. In *Mammals of Africa Volume IV: Hedgehogs, Shrews*, *and Bats* (eds M Happold, D Happold), pp. 375–378. London, UK: Bloomsbury Publishing.

30. Menzies JI. 1973 A study of leaf-nosed bats (*Hipposideros caffer* and *Rhinolophus landeri*) in a cave in northern Nigeria. *J. Mammal.* **54**, 930–945.

31. Mutere FA. 1970 The breeding biology of equatorial vertebrates: reproduction in the insectivorous bat, *Hipposideros caffer*, living at 0 27’N. *Bijdr. Dierkd.* **40**, 56–58.

32. Mutere FA. 1968 Breeding cycles in tropical bats in Uganda. *J* *Ecol* **56,** 5-9.

33. Anciaux de Faveaux M. 1978 Les cycles annuels de reproduction chez les Chiroptères cavernicoles du Shaba (S-E Zaïre) et du Rwanda. *Mammalia* **42**, 453–490.

34. Bernard R, Meester J. 1982 Females reproduction and female reproductive cycle of *Hipossideros caffer caffer* (Sundevall, 1846) in Natal, South Africa. *Ann. Transvaal. Mus.* **33**, 131–144.

35. Howell KM. 1976 An ecological study of three species of insectivorous bats near Kisarawe, Tanzania. *Unpublished PhD thesis, University of Dar es Salaam,*

36. Happold M. 2013 Hipposideros ruber Noack’s Leaf-nosed Bat. In *Mammals of Africa Volume IV: Bats and Shrews* (eds M Happold, H David), pp. 393–395.

37. Jones C. 1971 The bats of Rio Muni, West Africa. *J. Mammal.* **52**, 121–140.

38. Russo D, Maglio G, Rainho A, Meyer CFJ, Palmeirim JM. 2011 Out of the dark: Diurnal activity in the bat Hipposideros ruber on São Tomé island (West Africa). *Mammalian Biology - Zeitschrift für Säugetierkunde* **76**, 701–708.

39. Kingdon J. 1974 East African Mammals; an Atlas of Evolution in Africa. Volume II Part A (Insectivores and Bats).

40. Bergmans W, Hutson AM, Mickleburgh S, Monadjem A. *Lissonycteris angolensis*. The IUCN Red List of Threatened Species 2017. See http://www.iucnredlist.org/details/44698/0.

41. Happold D, Happold M. 1978 The fruit bats of Western Nigeria. 3. *Nigerian field* **43**, 30-37 .

42. Lavrenchenko LA, Kruskop SV, Morozov PN. 2004 Notes on the bats (Chiroptera) collected by the joint Ethiopian-Russian biological expedition, with remarks on their systematics, distribution, and ecology. *Bonn. Zool. Beitr.* **52**, 127–147.

43. Verschuren J. 1976 Les cheiropteres du Mont Nimba (Liberia). *Mammalia* **40**, 615–632.

44. Wolton RJ, Arak PA, Godfray H, Wilson RP. 1982 Ecological and behavioural studies of the megachiroptera at Mount Nimba, Liberia, with notes on microchiroptera. *Mammalia* **46**, 419–448.

45. Coe M. 1975 Mammalian ecological studies on Mount Nimba, Liberia. *Mammalia* **39**, 523–588.

46. Eisentraut M. 1964 La faune de chiroptères de Fernando-Po. *Mammalia* **28**, 529–552.

47. Adam J-P, Le Pont F. 1974 Les chiroptères cavernicoles de la République Populaire du Congo: notes bioécologiques et parasitologiques. *Annales Spéléologie* **29**, 143–154.

48. Happold M. 2013 *Tadarida condylura* Angolan free-tailed bat. In *Mammals of Africa Volume IV: Hedgehogs, Shrews*, *and Bats* (eds M Happold, D Happold), pp. 528–530. London, UK: Bloomsbury Publishing.

49. Brosset A. 1966 Les Chiroptères du Haut-Ivindo (Gabon). *Biologica Gabonica* **2**, 47–86.

50. BaagØe HJ. 1978 Observations on the biology of the banana bat, *Pipistrellus nanus*. In *Proceedings of the fourth international bat research conference* (eds RJ Olembo, JB Castelino, and FA Mutere*.). Kenya Literature Bureau, Nairobi*, pp. 275–282.

51. Stanley WT, Goodman SM. 2011 Small mammal inventories in the East and West Usambara and South Pare Mountains, Tanzania. 3. Chiroptera. *Fieldiana Life Earth Sci*. **4**, 34–52.

52. Kityo R, Kerbis JC. 1996 Observations on the distribution and ecology of bats in Uganda. *J. East Afr. Nat. Hist.* **85**, 49–63.

53. Anciaux de Faveaux M. 1983 Les cycles annuels de reproduction chez les chiropteres phytophiles au Shaba (SE Zaire) et au Rwanda. *Koninklijk Museum Voor Midden-Afrika Tervuren Belgie Annalen Zoologische Wetenschappen* **237**, 27–34.

54. O’Shea TJ. 1980 Roosting, social organization and the annual cycle in a Kenya population of the bat *Pipistrellus nanus*. *Zeitschrift für Tierpsychologie* **53**, 171–195.

55. Happold M, Others. 2013 *Pipistrellus nanus* Banana Pipistrelle (Banana Bat). In *Mammals of Africa Volume IV-Hedgehogs, Shrews and Bats*, Bloomsbury.

56. O’Shea TJ. 1977 Aspects of social organization, behavior and ecology in a Kenya population of the bat *Pipistrellus nanus*. *Unpublished* *PhD thesis*, Northern Arizona University, Arizona, US.

57. Bernard R, Happold M. 2013 *Nycteris thebaica* Egyptian slit-faced bat. In *Mammals of Africa Volume IV: Hedgehogs, Shrews*, *and Bats* (eds M Happold, D Happold), pp. 457--460. London, UK: Bloomsbury Publishing.

58. Bernard R. 1982 Female reproductive cycle of *Nycteris tbebaica* (Microchiroptera) from Natal, South Africa. *Z. Säugetierk* **47**, 12–18.

59. Mutere FA. 1968 The breeding biology of the fruit bat *Rousettus aegyptiacus* *E. Geoffroy* living at o degrees 0.22’S. *Acta Trop.* **25**, 97–108.

60. Okia NO. 1987 Reproductive cycles of East African bats. *J. Mammal.* **68**, 138–141.

61. Amman BR *et al.* 2012 Seasonal pulses of Marburg virus circulation in juvenile *Rousettus* *aegyptiacus* bats coincide with periods of increased risk of human infection. *PLoS Pathog.* **8**, e1002877.

62. Lučan RK *et al.* 2014 Reproductive seasonality of the Egyptian fruit bat (*Rousettus* *aegyptiacus*) at the Northern limits of its distribution. *J. Mammal.* **95**, 1036–1042.

63. Kulzer E. 1958 Untersuchungen über die biologie von flughunden der gattung *Rousettus* *Gray*. *Z. Morph. u. Okol. Tiere* **47**, 374–402.

64. Towner JS *et al.* 2009 Isolation of genetically diverse Marburg viruses from Egyptian fruit bats. *PLoS Pathog.* **5**, e1000536.

65. Penzhorn BL, Rautenbach IL. 1988 Reproduction of the Egyptian fruit bat *Rousettus* *aegyptiacus* in the Southern tropics. *S. Afr. J. Wildl. Res.* **18**, 88–92.

66. Kulzer E. 1962 Fledermäuse aus Tanganyika. *Z. Säugetierk* **27**, 164–181.

67. Ansell WFH. 1986 Some chiroptera from south-central Africa. *Mammalia* **50**, 507–519.

68. Smithers RHN. 1971 The mammals of Botswana. Trustees Nat. Mus. Rhodesia, Salisbury.

69. Taylor P. 1998 The Smaller Mammals of KwaZulu− Natal. Durban, South Africa: University of KwaZulu-Natal Press.

70. Benda P, Vallo P. 2009 Taxonomic revision of the genus *Triaenops* (chiroptera: hipposideridae) with description of a new species from Southern Arabia and definitions of a new genus and tribe. *Folia Zool*. **58**, 1–45.

71. Anciaux de Faveaux M. 1972 Répartition biogéographique et cycles annuels des chiropteres d’Afrique Centrale. *Unpublished PhD thesis*. University of Paris, Paris, France.

72. Mainoya JR. 1979 Spermatogenic and frontal sac gland activity in *Triaenops persicus* (Chiroptera: Hipposideridae). *Afr. J. Ecol.* **17**, 127–129.
