# Supplementary material for: Reproduction of East-African bats may guide risk mitigation for coronavirus spillover
Source: One Health Outlook. 2020 Feb 7;2:2. doi: 10.1186/s42522-019-0008-8 (PMC7149079; doi:10.1186/s42522-019-0008-8)
Supplement: Supplementary file 2 — Additional file 2: Bibliographic references for the traits of the bat species included in the study. [file 42522_2019_8_MOESM2_ESM.docx]

**Additional file 2**

Bibliographic references for the traits of the bat species included in the study.

***Chaerephon pumilus***

- Bouchard, Sylvie. 1998 *Chaerephon pumilus*. *Mammalian Species*. **574**: 1–6.
- Happold DCD, Happold M. 1990 Reproductive strategies of bats in Africa. *J. Zool.* **222**, 557–583.
- Happold DC, Happold M. 1989 Reproduction of Angola free-tailed bats (*Tadarida condylura*) and little free-tailed bats (*Tadarida pumila*) in Malawi (Central Africa) and elsewhere in Africa. *J. Reprod. Fertil.* **85**, 133–149.
- Happold M, Happold D, editors. 2013 *Mammals of Africa Volume IV-Hedgehogs, Shrews and Bats*. London, UK: Bloomsbury Publishing.
- Koopman KF, Mumford RE, Heisterberg JF. 1978 Bat records from Upper Volta, West Africa. Am. Mus. Novit. **2643**, 1- 6.
- Marshall AJ, Corbet PS. 1959 The breeding biology of equatorial vertebrates: reproduction of the bat *Chaerephon hindei* Thomas at latitude 0 26′ N. *Proc. Zool. Soc. Lond.* ***132***, 607–616.
- Mcwilliam AN. 1987 Polyoestry and postpartum oestrus in *Tadarida* (*Chaerephon*) *pumila* (Chiroptera: Molossidae) in Northern Ghana, West Africa*. J. Zool*. **213**, 735–39.
- McWilliam AN. 1988 Social organisation of the bat *Tadarida* (*Chaerephon*) *pumila* (Chiroptera: Molossidae) in Ghana, West Africa. *Ethology*. **77**, 115–124.
- Mickleburgh S, Hutson AM, Racey PA, Ravino J, Bergmans W, Cotterill F, Gerlach J. 2014 *Chaerephon pumilus*. *The* IUCN Red List of Threatened Species. See http://www.iucnredlist.org/details/4317/0.
- Mutere FA. 1973 Reproduction in two species of equatorial free-tailed bats (Molossidae). *Afr. J. Ecol.* **11**, 271–280.
- Van der Merwe M, Giddings SR, Rautenbach and IL. 1987 Post-partum oestrus in the little free-tailed bat, *Tadarida* (*Chaerephon*) *pumila* (Microchiroptera: Molossidae) at 24° S. *J. Zool*. **213**, 317–26.
- Van der Merwe M, Rautenbach IL, Van der Colf WJ. 1986 Reproduction in females of the little free-tailed bat, *Tadarida* (*Chaerephon*) *pumila*, in the eastern Transvaal, South Africa. *J. Reprod. Fertil.* **77**, 355–364.

***Coleura afra***

- Anciaux de Faveaux M. 1973 Essai de synthese sur la réproduction de chiropteres d’Afrique (Région Faunistique Ethiopienne). *Period. Biol.* **75**, 195–199.
- Bernard RT, Cumming GS. 1997 African bats: evolution of reproductive patterns and delays. *Q. Rev. Biol.* **72**, 253–274.
- Cardiff SG, Ratrimomanarivo FH. 2009 Hunting, disturbance and roost persistence of bats in caves at Ankarana, Northern Madagascar. *Afr. J. Ecol*. **47**, 640–649.
- Dunlop J. 1997 *Coleura Afra*. *Mammalian Species.* **566**, 1–4.
- Kingdon J. 1974 *East African Mammals; an Atlas of Evolution in Africa*. Volume II Part A: Insectivores and Bats. Chicago, Illinois, US: University of Chicago Press.
- Kock D. 1969 Die Fledermaus-Fauna des Sudan. *Abh. Senckenberg. Naturf. Ges*. **521,** 1-23.
- Makori B. 2015 Survey and conservation of cave-dwelling bats in coastal Kenya. Karatina University, School of Natural Resources and Environmental Studies.
- Matthews LH. 1942 Notes on the genitalia and reproduction of some African rats. *Proc. Zool. Soc. Lond.* B, **111**, 289–342.
- McWilliam AN. 1987 The reproductive and social biology of *Coleura afra* in a seasonal environment. In *Recent Advances in the Study of Bats* (eds MB Fenton, P Racey, and MV Rayner,), pp 324-350. Cambridge, UK: Cambridge University Press.

***Eidolon helvum***

- Bergmans W. 1990 Taxonomy and biogeography of African fruit bats (Mammalia, Megachiroptera). 3. The genera *Scotonycteris* Matschie, 1894, *Casinycteris* Thomas, 1910, *Pteropus* Brisson, 1762, and *Eidolon* Rafinesque, 1815.” *Beaufortia*. **40,** 111–77.
- DeFrees SL, Wilson DE. 1988 *Eidolon Helvum*. *Mammalian Species*. **312**, 1–5.
- Fayenuwo JO, Halstead LB. 1974 Breeding cycle of straw-colored fruit bat, *Eidolon* *Helvum*, at Ile-Ife, Nigeria. *J. Mammal.* **55,** 453–454.
- Funmilayo O. 1979 Ecology of the straw- coloured fruit bat in Nigeria. *Revue Zool. Africaine*, **93**, 589–600.
- Huggel-Wolf H, Huggel-Wolf ML. 1965 La biologie d’ “*Eidolon helvum*” (Kerr) (Megachiroptera). *Acta tropica* **22**, 1–10.
- Jones C. 1972 Comparative ecology of three pteropid bats in Rio Muni, West Africa. J. Zool. **167**, 353–70.
- Kingdon J. 1974 *East African Mammals; an Atlas of Evolution in Africa*. Volume II Part A: Insectivores and Bats. Chicago, Illinois, US: University of Chicago Press.
- Malbrant R, Maclatchy A. 1949 *Faune de L’équateur Africain Français*. Paris, France: P. Lechevalier.
- Mickleburgh S, Hutson AM, Bergmans W, Fahr J, Racey PA. 2010 *Eidolon Helvum*. The IUCN Red List of Threatened Species. See https://www.iucnredlist.org/species/7084/12824968
- Mutere, FA. 1965 Delayed implantation in an Equatorial fruit bat. *Nature*. **207**, 780.
- Mutere, FA. 1967 The breeding biology of equatorial vertebrates: reproduction in the fruit bat, *Eidolon helvum*, at latitude 0 20′ N. *J. Zool.* **153**, 153–61.
- Mutere, FA, Wilson D, Gardner A. 1980 *Eidolon helvum* revisited. In *Proceedings of the 5th International Bat Research Conference* (eds Wilson DE, Gardner AL), pp 145-150. Lubbock, Texas, US: Texas Tech Press.
- Nowak J, Paradiso JL. 1983. *Walker’s mammals of the world*. Baltimore, Maryland, US: Johns Hopkins University Press.
- Ossa G, Kramer-Schadt S, Peel AJ, Scharf AK, Voigt CC. 2012 The movement ecology of the straw-colored fruit bat, *Eidolon Helvum*, in sub-saharan Africa assessed by stable isotope ratios. *PloS One* **7**. e45729.
- Peel, AJ. 2012 The Epidemiology of Lagos Bat virus and henipaviruses in straw-coloured fruit bats (*Eidolon Helvum*), using population genetics to infer population connectivity. *Unpublished PhD thesis*. University of Cambridge, Cambridge, UK.
- Peel AJ *et al.* 2017 How does Africa’s most hunted bat vary across the continent? Population traits of the straw-coloured fruit bat (*Eidolon helvum*) and its interactions with humans. *Acta Chiropt.* **19**, 77–92.
- Perpetra A., Kityo M. 2009 Populations of *Eidolon helvum* in Kampala over 40 years. Journal of Forestry and Nature Conservation **79**, 1–7.
- Richter HV, Cumming GSC. 2006 Food availability and annual migration of the straw-colored fruit bat (*Eidolon helvum*). *J. Zoology*. **268**, 35–44.
- Richter HV, Cumming GSC. 2008 First application of satellite telemetry to track African straw-coloured fruit bat migration. *J. Zool.* **275**, 172–76.
- Rosevear DR. 1965 *The bats of West Africa*. London, UK: The British Museum.
- Thomas DW. 1983 The annual migrations of three species of West African fruit bats (Chiroptera: Pteropodidae). *Can J Zool*. **61**, 2266–72.

***Hipposideros sp.***

- Anciaux de Faveaux M. 1978 Les cycles annuels de reproduction chez les Chiroptères cavernicoles du Shaba (S-E Zaïre) et du Rwanda. *Mammalia* **42**, 453–490
- Bell, Gary P. 1987 Evidence of a harem social system in *Hipposideros caffer* (chiroptera: hipposideridae) in Zimbabwe. *J. Trop. Ecol*. **3**, 87–90.
- Bernard R, Meester J. 1982 Females reproduction and female reproductive cycle of *Hipossideros caffer caffer* (Sundevall, 1846) in Natal, South Africa. *Ann. Transvaal. Mus.* **33**, 131–144.
- Bowie RCK, Jacobs DS, Taylor PJ. 1999 Resource use by two morphologically similar insectivorous bats (*Nycteris thebaica* and *Hipposideros caffer*). *S. Afr. J. Zool.* **34**, 27–33.
- Churchill S, Draper R, Marais E. 1997 Cave utilisation by Namibian bats: population, microclimate and roost selection. S. Afr. J. Wildl. Res. **27**, 44 – 50.
- Cotterill, Fenton Peter David. 2001 Notes on mammal collections and biodiversity conservation in the Ikelenge pedicle, Mwinilunga district, Northwest Zambia. *Occasional Publications in Biodiversity*, **10**. Biodiversity Foundation for Africa. Famona, Bulawayo, Zimbabwe.
- Dunning DC, Krüger M. 1996 Predation upon moths by free-foraging *Hipposideros* *caffer*.” *Mammalogy* **77**, 708–15.
- Fenton MB, Bell GP, Thomas DW. 1980 Echolocation and feeding behaviour of *Taphozous mauritianus* (Chiroptera: Emballonuridae). *Can. J. Zool.* **58**, 1774–77.
- Fenton, MB, Boyle NGH, Harrison TM, Oxley DJ. 1977 Activity patterns, habitat use, and prey selection by some African insectivorous bats. *Biotropica*. **9**: 73–85.
- Heller, K. G. 1992 The echolocation calls of *Hipposideros ruber and Hipposideros caffer*. In: Prague Studies in Mammalogy (eds Horácek I, Vohralík V), pp. 75-77. Praha, Czech Republic: Charles University Press.
- Jacobs DS. 2000 Community level support for the allotonic frequency hypothesis. *Acta Chiropt*. **2**, 197–207.
- Jones C. 1971 The bats of Rio Muni, West Africa. *J. Mammal.* **52**, 121–140.
- Kingdon J. 1974 *East African Mammals; an Atlas of Evolution in Africa*. Volume II Part A: Insectivores and Bats. Chicago, Illinois, US: University of Chicago Press.
- Menzies JI. 1973 A study of leaf-nosed bats (*Hipposideros caffer* and *Rhinolophus landeri*) in a cave in northern Nigeria. *J. Mammal.* **54**, 930–945.
- Mutere FA. 1968 Breeding cycles in tropical bats in Uganda. *J* *Ecol* **56,** 5-9.
- Whitaker O, Black H. 1976 Food habits of cave bats from Zambia, Africa. *Mammalogy* **57**,199–204.
- Wright GS. 2009 *Hipposideros caffer* (Chiroptera: Hipposideridae). *Mammalian Species*, **845**, 1-9.
- Anciaux de Faveaux M. 1978 Les cycles annuels de reproduction chez les Chiroptères cavernicoles du Shaba (S-E Zaïre) et du Rwanda. *Mammalia* **42**, 453–490.
- Bernard RT, Cumming GS. 1997 African bats: evolution of reproductive patterns and delays. *Q. Rev. Biol.* **72**, 253–274.
- Howell KM. 1976 An ecological study of three species of insectivorous bats near Kisarawe, Tanzania. *Unpublished PhD thesis,* University of Dar es Salaam, Dar es Salaam, Tanzania.
- Jones, G, Morton M, Hughes PM, Budden RM. 1993 Echolocation, flight morphology and foraging strategies of some West African Hipposiderid bats” *J. Zool.* **230**, 385–400.
- Kingdon J. 1974 *East African Mammals; an Atlas of Evolution in Africa*. Volume II Part A: Insectivores and Bats. Chicago, Illinois, US: University of Chicago Press.
- Kityo R, Kerbis JC. 1996 Observations on the distribution and ecology of bats in Uganda. *J. East Afr. Nat. Hist.* **85**, 49–63.
- Russo D, Maglio G, Rainho A, Meyer CFJ, Palmeirim JM. 2011 Out of the dark: diurnal activity in the bat *Hipposideros ruber* on São Tomé island (West Africa). *Mammalian Biology* **76**, 701–708.
- Verschuren J. 1957 Ecologie, biologie et systematique des cheiropteres. Exploration du Parc National de la Garamba; *Inst. Parcs Nat. Congo*, Brussels, Belgium.

***Lissonycteris angolensis***

- Anciaux de Faveaux M. 1978 Les cycles annuels de reproduction chez les Chiroptères cavernicoles du Shaba (S-E Zaïre) et du Rwanda. *Mammalia* **42**, 453–490.
- Happold M, Happold D, editors. 2013 *Mammals of Africa Volume IV-Hedgehogs, Shrews and Bats*. London, UK: Bloomsbury Publishing.
- Kingdon J. 1974 *East African Mammals; an Atlas of Evolution in Africa*. Volume II Part A: Insectivores and Bats. Chicago, Illinois, US: University of Chicago Press.
- Happold D, Happold M. 1978 The fruit bats of Western Nigeria. 3. *Nigerian field* **43**, 30-37 .
- Wolton RJ, Arak PA, Godfray H, Wilson RP. 1982 Ecological and behavioural studies of the Megachiroptera at Mount Nimba, Liberia, with notes on Microchiroptera. *Mammalia* **46**, 419–448.
- Coe M. 1975 Mammalian ecological studies on Mount Nimba, Liberia. *Mammalia* **39**, 523–588.
- Jones ML. 1982 Longevity of captive mammals. *Zool. Gart.* **52**, 113–128.
- Adam J-P, Le Pont F. 1974 Les chiroptères cavernicoles de la République Populaire du Congo: notes bioécologiques et parasitologiques. *Annales Spéléologie* **29**, 143–154.
- Lavrenchenko LA, Kruskop SV, Morozov PN. 2004 Notes on the bats (Chiroptera) collected by the joint Ethiopian-Russian biological expedition, with remarks on their systematics, distribution, and ecology. *Bonn. Zool. Beitr.* **52**, 127–147.
- Verschuren J. 1976 Les cheiropteres du Mont Nimba (Liberia). *Mammalia* **40**, 615–632.
- Eisentraut M. 1964 La faune de chiroptères de Fernando-Po. *Mammalia* **28**, 529–552.
- Bergmans W, Hutson AM, Mickleburgh S, Monadjem A. *Lissonycteris angolensis*. The IUCN Red List of Threatened Species 2017. See http://www.iucnredlist.org/details/44698/0.

***Mop condylurus***

- Happold, DCD, Happold M. 1988 Renal form and function in relation to the ecology of bats (chiroptera) from Malawi, Central Africa. *J. Zool.* **215**, 629–55.
- Happold, DCD, Happold M, Hill JE. 1987 The bats of Malawi. *Mammalia*, **51**, 337–414.
- Happold, DCD, Happold M. 1989 Reproduction of Angola free-tailed bats (*Tadarida Condylura*) and little free-tailed bats (*Tadarida Pumila*) in Malawi (Central Africa) and elsewhere in Africa. *J. Reprod, Fertil*. **85**, 133–149.
- Happold M, Happold D, editors. 2013 *Mammals of Africa Volume IV-Hedgehogs, Shrews and Bats*. London, UK: Bloomsbury Publishing.
- Happold M. 2013 *Tadarida condylura* Angolan free-tailed bat. In *Mammals of Africa Volume IV: Hedgehogs, Shrews*, *and Bats* (eds M Happold, D Happold), pp. 505–507. London, UK: Bloomsbury Publishing.
- Mutere FA. 1973 Reproduction in two species of equatorial free-tailed bats (Molossidae). *Afr. J. Ecol.* **11**, 271–280.
- Noer CL, Dabelsteen T, Bohmann K, Monadjem A. 2012 Molossid bats in an African agro-ecosystem select sugarcane fields as foraging habitat. *Afric. Zool.* **47**, 1–11.
- O’Shea TJ, Vaughan TA. 1980 Ecological observations on an East African bat community. *Mammalia*. **44**, 485–496.
- Vivier L, van der Merwe M. 2007 The incidence of torpor in winter and summer in the angolan free-tailed bat, *Mops condylurus* (Microchiroptera: Molossidae), in a subtropical environment, Mpumulanga, South Africa. *Afric. Zool.* **42,** 50–58.
- Whitaker JO, Black H. 1976 Food habits of cave bats from Zambia, Africa. *J. Mammal.* **57**, 199–204.
- Whitaker JO, Mumford RE. 1978. Foods and ectoparasites of bats from Kenya, East Africa. *J. Mammal*. **59**, 632–34.

***Neoromicia nana***

- Anciaux de Faveaux M. 1983 Les cycles annuels de reproduction chez les chiropteres phytophiles au Shaba (SE Zaire) et au Rwanda. *Ann. Mus. R. Afr. Centr. Sci. Zool*. **237**, 27–34.
- Bernard RTF. Happold DCD, Happold M. 1997 Sperm storage in a seasonally reproducing African vespertilionid, the banana bat (*Pipistrellus nanus*) from Malawi. *J. Zool.* **241**, 161–74.
- Brosset, A. 1966 Les chiroptères du Haut-Ivindo (Gabon). Biologica Gabonica **2**, 47–86.
- Happold, DCD, Happold M. 1990 The domiciles, reproduction, social organisation and sex ratios of the banana bat *Pipistrellus nanus* (chiroptera, vespertilionidae) in Malawi, Central Africa.” *Z. Säugetierkd*. **55**, 145–60.
- Happold M, Others. 2013 *Pipistrellus nanus* banana pipistrelle (banana bat). In *Mammals of Africa Volume IV-Hedgehogs, Shrews and Bats*, Bloomsbury.
- Kityo R, Kerbis JC. 1996 Observations on the distribution and ecology of bats in Uganda. *J. East Afr. Nat. Hist.* **85**, 49–63.
- Lausen, CL, Barclay RMR. 2005 *Pipistrellus nanus*. *Mammalian species*, **784**, 1–7.
- LaVal RK, LaVal ML. 1977 Reproduction and behavior of the African banana bat, *Pipistrellus nanus*. *J. Mammal.* **58**, 403–10.
- O’Shea TJ. 1977 Aspects of social organization, behavior and ecology in a Kenya population of the bat *Pipistrellus nanus*. *Unpublished* *PhD thesis*, Northern Arizona University, Arizona, US.
- O’Shea TJ. 1980 Roosting, social organization and the annual cycle in a Kenya population of the bat *Pipistrellus nanus*. *Zeitschrift für Tierpsychologie* **53**, 171–195.
- O’Shea TJ, Vaughan TA. 1980 Ecological observations on an East African bat community. *Mammalia* **44**, 485–496.

Stanley WT, Goodman SM. 2011 Small mammal inventories in the East and West Usambara and South Pare Mountains, Tanzania. 3. Chiroptera. *Fieldiana Life Earth Sci*. **4**, 34–52.

- Stanley WT, Goodman SM, Kihaule PM, Howell KM. 2000 A survey of the small mammals of the Gonja Forest Reserve, Tanzania. *J. East Afr. Nat. Hist*. **89**, 73–83.
- Van Der Merwe, M, Stirnemann RL. 2007 Reproduction of the banana bat, *Neoromicia nanus*, in Mpumalanga Province, South Africa, with a discussion on sperm storage and latitudinal effects on reproductive strategies. S. Afr. J. Wild. Res. **37**, 53–60.

***Nycteris cf. thebaica***

- Anciaux de Faveaux M. 1978 Les cycles annuels de reproduction chez les Chiroptères cavernicoles du Shaba (S-E Zaïre) et du Rwanda. *Mammalia* **42**, 453–490.
- Bernard R. 1982 Female reproductive cycle of *Nycteris tbebaica* (Microchiroptera) from Natal, South Africa. *Z. Säugetierk* **47**, 12–18.
- Bernard, RTF, Happold M. 2013 *Nycteris Thebaica* Egyptian slit-faced bat. In *Mammals of Africa Volume IV: Hedgehogs, Shrews*, *and Bats* (eds M Happold, D Happold), pp. 457-460. London, UK: Bloomsbury Publishing.
- Chapman RF. 1958 Some observations on the food of a bat. *Ann. Mag. Nat. Hist.* **1**, 188–192.
- Aldridge HDJN, Obrist M, Merriam HG, Fenton MB. 1990 Roosting, vocalizations, and foraging by the African bat*, Nycteris thebaica*. *J. Mammalogy*. **71**, 242–46.
- LaVal RK, LaVal ML. 1980 Prey selection by the slit-faced bat *Nycteris thebaica* (Chiroptera: Nycteridae) in Natal, South Africa. *Biotropica*. **12**, 241–46.
- Whitaker O, Black H. 1976 Food habits of cave bats from Zambia, Africa. *Mammalogy* **57**,199–204.

***Pipistrellus cf. hesperidus***

- Happold, DCD, Happold M. 1988 Renal form and function in relation to the ecology of bats (chiroptera) from Malawi, Central Africa. *J. Zool.* **215**, 629–55.
- Happold, DCD, Happold M, Hill JE. 1987 The bats of Malawi. *Mammalia*, **51**, 337–414.

Happold M, Happold D, editors. 2013 *Mammals of Africa Volume IV-Hedgehogs, Shrews and Bats*. London, UK: Bloomsbury Publishing.

- Smithers RHN. 1971 The mammals of Botswana. Trustees Nat. Mus. Rhodesia, Salisbury.
- Smithers RHN, Wilson VJ. 1979 Check list and atlas of the mammals of Zimbabwe Rhodesia. Trustees Nat. Mus. Rhodesia, Salisbury.

***Rhinolophus cf. clivosus***

- Baeten, B, Van Cakenberghe V, De Vree F. 1984 An annotated inventory of a collection of bats from Rwanda.” *Revue Zool. Afr.* **98**, 183–96.
- Benda P, Vallo P. 2012. New Look on the Geographical Variation in *Rhinolophus Clivosus* with description of a new horseshoe bat species from Cyrenaica, Libya. *Vespertilio*. **16.** 69–96.
- Bernard RTF. 1983 Reproduction of *Rhinolophus clivosus* (Microchiroptera) in Natal, South Africa. *Z. Säugetierkd*. **48**, 321–29.
- Bernard RTF. 1988. Prolonged sperm storage in male cape horseshoe bats. *Naturwissenschaften* **75**, 213–14.
- Churchill S, Draper R, Marais E. 1997 Cave utilisation by Namibian bats: population, microclimate and roost selection. S. Afr. J. Wildl. Res. **27**, 44 – 50.
- Happold, DCD, Happold M. 1988 Renal form and function in relation to the ecology of bats (chiroptera) from Malawi, Central Africa. *J. Zool.* **215**, 629–55.
- Happold M, Others. 2013 *Pipistrellus nanus* banana pipistrelle (banana bat). In *Mammals of Africa Volume IV-Hedgehogs, Shrews and Bats*, Bloomsbury.
- McDonald J, Rautenbach JT, Others. 1990 Roosting requirements and behaviour of five bat species at de hoop guano cave, Southern Cape Province of South Africa. S African J Wildl Res. **20**, 157–161.
- Rautenbach, IL 1982 The mammals of the Transvaal. *Ecoplan* **1**, Pretoria, South Africa.
- Stoffberg SM, Schoeman C, Matthee CA. 2012 Correlated genetic and ecological diversification in a widespread Southern African horseshoe bat.” *PloS One* **7**: e31946.

***Taphozous mauritanus***

- Anciaux de Faveaux M. 1983 Les cycles annuels de reproduction chez les chiropteres phytophiles au Shaba (SE Zaire) et au Rwanda. *Ann. Mus. R. Afr. Centr. Sci. Zool*. **237**, 27–34.
- Dengis CA. 1996 *Taphozous mauritianus*. *Mammalian Species*. **522**, 1-5.
- Fenton MB, Bell GP, Thomas DW. 1980 Echolocation and feeding behaviour of *Taphozous mauritianus* (Chiroptera: Emballonuridae). *Can. J. Zool.* **58**, 1774–77.
- Happold, DCD, Happold M. 1988 Renal form and function in relation to the ecology of bats (chiroptera) from Malawi, Central Africa. *J. Zool.* **215**, 629–655.
- Happold DCD, Happold M. 1990 Reproductive strategies of bats in Africa. *J. Zool.* **222**, 557–583.
- Happold, DCD, Happold M, Hill JE. 1987 The bats of Malawi. *Mammalia*, **51**, 337–414.
- Kingdon J. 1974 *East African Mammals; an Atlas of Evolution in Africa*. Volume II Part A: Insectivores and Bats. Chicago, Illinois, US: University of Chicago Press.
- O’Shea TJ, Vaughan TA. 1980 Ecological observations on an East African bat community. *Mammalia*. **44**, 485–496.
- Smithers RHN. 1971 The mammals of Botswana. Trustees Nat. Mus. Rhodesia, Salisbury.
- Taylor P. 1998 The Smaller Mammals of KwaZulu− Natal. Durban, South Africa: University of KwaZulu-Natal Press.
- Taylor P. Cheney JC, Sapsford C. 1999 Roost habitat evaluation and distribution of bats (chiroptera) in the Durban metropolitan region. *Durban Mus. Nov*. **24**, 62–71.
- Verschuren, J. 1957. Ecologie, biologie et systematique des cheiropteres*. Explor. Parc. natn. Garamba Miss. H. deSaeger*. **7**, 1-473.

***Triaenops persicus***

- Anciaux de Faveaux M. 1972 Répartition biogéographique et cycles annuels des chiropteres d’Afrique Centrale. *Unpublished PhD thesis*. University of Paris, Paris, France.
- Benda P, Vallo P. 2009 Taxonomic revision of the genus *Triaenops* (chiroptera: hipposideridae) with description of a new species from Southern Arabia and definitions of a new genus and tribe. *Folia Zool*. **58**, 1–45.
- Benda, P, Faizolâhi K, Andreas M, Obuch J. 2012 Bats (Mammalia: Chiroptera) of the Eastern Mediterranean and Middle East. Part 10. Bat Fauna of Iran. *Acta Soc. Zool. Bohem*. **76**, 163–562.
- Happold M, Happold D, editors. 2013 *Mammals of Africa Volume IV-Hedgehogs, Shrews and Bats*. London, UK: Bloomsbury Publishing.
- Howell KM. 1976 An ecological study of three species of insectivorous bats near Kisarawe, Tanzania. *Unpublished PhD thesis,* University of Dar es Salaam, Dar es Salaam, Tanzania.
- Mainoya, JR. 1979 Spermatogenic and frontal sac gland activity in *Triaenops persicus* (Chiroptera: Hipposideridae). *Afr. J. Ecol.* **17**, 127–29.
- Smithers, RHN. 1983. *The Mammals of the Southern African Sub-region*. Cambridge, UK: Cambridge University Press.
