# Supplementary material for: Reproduction of East-African bats may guide risk mitigation for coronavirus spillover
Source: One Health Outlook. 2020 Feb 7;2:2. doi: 10.1186/s42522-019-0008-8 (PMC7149079; doi:10.1186/s42522-019-0008-8)
Supplement: Supplementary file 3 — Additional file 3: Imputation of the reproductive season to those bats whose biology is insufficiently known. Model methods, results, and literature cited. Table S3. Summary of the coefficients’ posterior probability distributions of the selected model for the imputation of the reproductive seasons of bats missing this data. Figure S3.1. The discrepancy between the observed (inferred) period and the period predicted by the selected imputation model considering 5 thousand posterior predictions of the observed period. Values of zero indicate matching, (observed period - predicted period). Figure S3.2. Left: the proportion of bats in each period after the imputation for those bats with the non-inferred season. Each line connects the proportion of individuals per reproductive season in each of the 5,000 Markov Chain Monte Carlo iteration. Right: the distribution of the crude coronavirus detection per reproductive season across the 5,000 Markov Chain Monte Carlo sampling iterations after imputing the periods when un-inferred. The black boxplots show the distribution of the coronavirus detection per period, while the light and dark colored boxes above and below show the interquartile detection in non-adults and adult bats, respectively, per period. [file 42522_2019_8_MOESM3_ESM.docx]

**Additional file 3**

Imputation of the reproductive season to those bats whose biology is insufficiently known. Model methods, results, and literature cited.

**Description of the imputation model**

Because it was not feasible to allocate the *Pipistrellus hesperidus*, *Rhinolophus cf. clivosus*, and *Lissonycteris angolensis* into a reproductive season we assumed that their reproductive seasons were seasonal and that these seasons were “Missing at Random”. Under these assumptions we imputed the corresponding reproductive season during the Markov Chain Monte Carlo process. The imputation allowed us to include all samples in model fitting, which can be advantageous for bias and precision, and to include our uncertainty about the reproductive season for the unallocated bats. Specifically, the unknown probability, 𝜋*_i_*, that the *i*^th^ bat with unknown season was in the RW season at the time of sampling was linearly related to a series of covariates via a logit link:

logit(π*_i_*) = a + b_1_*x*_1_*_i_*  + ... + b*_m_ x_mi_*

We considered the following covariates to model the π*_i_*: historical monthly precipitation, latitude of the sampling event, as these factors have been associated with bat breeding [1], the day of the year of the sampling event, and the number of litters per year of the corresponding species because these covariates could trace the current season. We assumed that only these covariates could determine the reproductive season across all species and sampling events, consequently, the candidate imputation model did not contain clustering terms.

The monthly average precipitation for the last 20 years at the sampling location was estimated by averaging the precipitation accumulated per month, according to the high-spatial resolution (~4 km) dataset TerraClimate [2], during the previous 20 years. The historical precipitation was allocated to bats depending on the month of sampling.

As the NUTS sampler (STAN) sampled to estimate the PPDs of the general model, the 𝜋’s were estimated, and the bats with unknown reproductive season were allocated through a Bernoulli trial. We used the same priors described in the main text, whilst the convergence of the chains was evaluated as already described also. To select the final imputation model, we evaluated the goodness-of-fit by checking the discrepancy between the inferred reproductive season for the bat with inferred reproductive season and the imputed reproductive seasons, secondly, we quantified the WAIC (widely-applicable or Watanabe-Akaike information criteria) [3] and the PSIS LOO-CV (Pareto smoothed importance sampling leave-one-out cross-validation) [4] of each model using the loo function of the “loo” package v. 2.0 [5] for “R” [6] targeting models with low values. Finally, we followed the parsimonious principle to include the least number of covariates providing predictions comparable to the best performing model.

**Results of the imputation model and process**

The selected model to impute the reproductive season included as predictors the latitude at the sampling event, the day of the year of the sampling event, the number of litters per year of the corresponding species (1 litter per year versus more than one litter per year), and the historical precipitation for the month and location of the corresponding sampling event. The main properties of the PPDs of these covariates (mean, standard deviation and the 90% HPDI) are shown in Supplementary Table S3. The WAIC and the PSIS LOO-CV for the selected imputation model were equal to 764.3 (s.e. = 19.0). Less complex models had higher values for these indexes, while more complex models including two-way interaction terms, produced marginally lower WAIC and PSIS LOO-CV estimates, but they overlapped with the ones of the selected model. Parsimony criteria favored the model without interaction terms.

For those bats with inferred season the global discrepancy with respect to the predicted season (inferred seasons versus posterior predictive distribution of the imputation) was ~40% and at most ~55% when the inferred season was “Not recent weaning” (Supplementary Figure 3.1). However, the imputation of the un-inferred season did not impact the final proportion of individuals in either reproductive period over the sampling chains (Supplementary Figure 3.2, left). Similarly, the crude coronavirus detection and the crude detection per season per age category were not significantly impacted by the imputation process (Supplementary Figure 3.2, right).

**Table S3. Summary of the coefficients’ posterior probability distributions of the selected model for the imputation of the reproductive seasons of bats missing this data.**

| **Covariate** | **Mean** | **SD** | **90% HPDI** |
| --- | --- | --- | --- |
| Intercept | 0.462 | 0.053 | 0.374 - 0.549 |
| Day of the year | -0.501 | 0.059 | -0.600 - -0.408 |
| Latitude | 0.480 | 0.056 | 0.381 - 0.565 |
| Litters per year | 1.209 | 0.121 | 1.008 - 1.412 |
| Historical monthly precipitation | -0.018 | 1.988 | -3.320 - 3.036 |

SD = Standard deviation and 90% HPDI = 90% high posterior density interval.

**Figure S3.1. The discrepancy between the observed (inferred) period and the period predicted by the selected imputation model considering 5 thousand posterior predictions of the observed period. Values of zero indicate matching, (observed period - predicted period).**


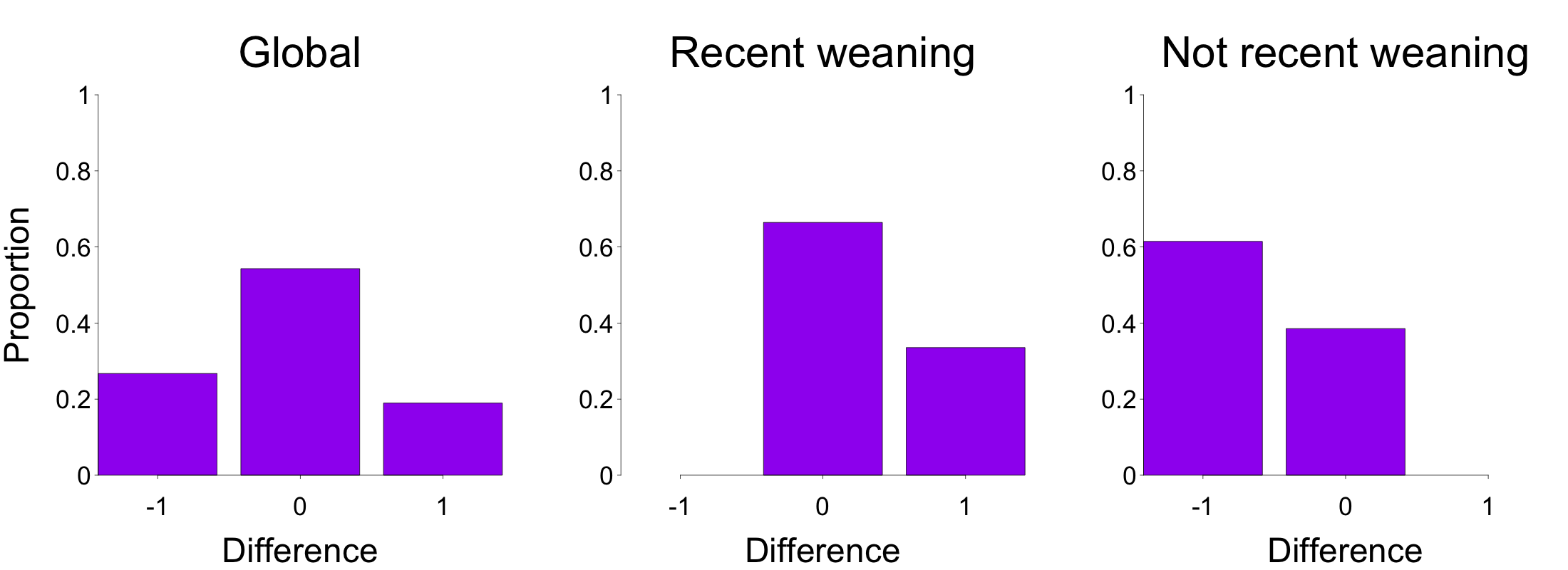


**Figure S3.2. Left: the proportion of bats in each period after the imputation for those bats with the non-inferred season. Each line connects the proportion of individuals per reproductive season in each of the 5,000 Markov Chain Monte Carlo iteration. Right: the distribution of the crude coronavirus detection per reproductive season across the 5,000 Markov Chain Monte Carlo sampling iterations after imputing the periods when un-inferred. The black boxplots show the distribution of the coronavirus detection per period, while the light and dark colored boxes above and below show the interquartile detection in non-adults and adult bats, respectively, per period.**

**
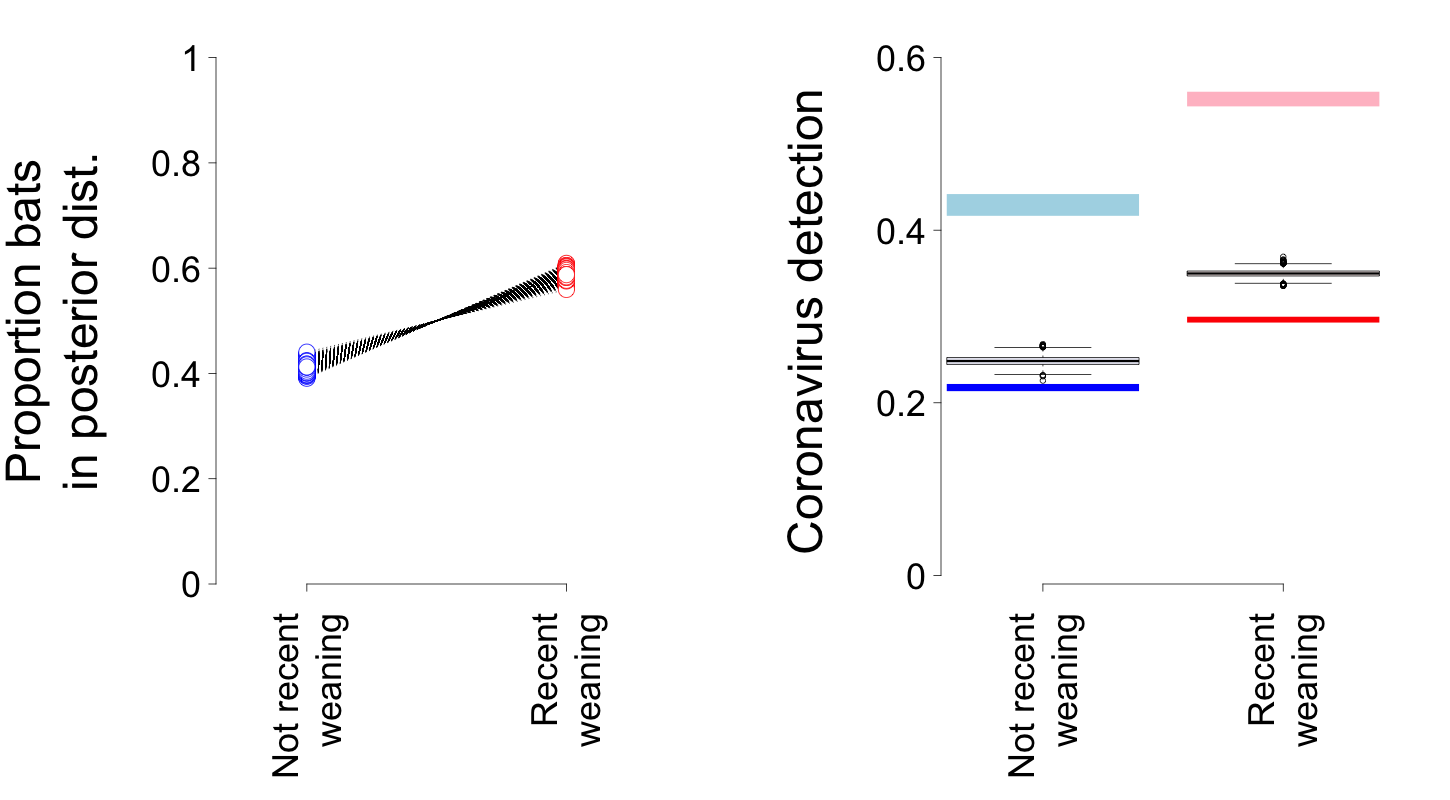
**

**Additional file 3 References**

1. Heideman PD. 2000 Environmental Regulation of Reproduction. In *Reproductive Biology of Bats* (eds EG Crichton, PH Krutzsch), pp. 469–499. London, UK: Academic Press.

2. Abatzoglou JT, Dobrowski SZ, Parks SA, Hegewisch KC. 2018 TerraClimate, a high-resolution global dataset of monthly climate and climatic water balance from 1958–2015. *Scientific Data* 5, 170191.

3. Watanabe S. 2010 Asymptotic equivalence of Bayes cross-validation and widely applicable information criterion in singular learning theory. *J. Mach. Learn. Res.* 11, 3571–3594.

4. Vehtari A, Gelman A, Gabry J. 2017 Practical Bayesian model evaluation using leave-one-out cross-validation and WAIC. *Stat. Comput.* 27, 1413–1432.

5. Vehtari A, Gelman A, Gabry J. 2016 loo: Efficient leave-one-out cross-validation and WAIC for Bayesian models. *R package version 0. 1*. *6*.

6. R Core Team. 2018 R: A language and environment for statistical computing. Vienna, Austria. *URL http://www. R-project. org*
