# Supplementary material for: Reproduction of East-African bats may guide risk mitigation for coronavirus spillover
Source: One Health Outlook. 2020 Feb 7;2:2. doi: 10.1186/s42522-019-0008-8 (PMC7149079; doi:10.1186/s42522-019-0008-8)
Supplement: Supplementary file 4 — Additional file 4: Table S4. Summary of the Alpha- and Betacoronaviruses (alphaCoV and betaCoV, respectively) found in the microbats tested. [file 42522_2019_8_MOESM4_ESM.docx]

**Additional file 4**

**Table A4. Summary of the Alpha- and Betacoronaviruses (alphaCoV and betaCoV, respectively) found in the microbats tested.**

| **Species** | **Positive individuals for alphaCoV** | **Positive individuals for betaCoV** | **alphaCoV found** | **betaCoV found** |
| --- | --- | --- | --- | --- |
| *Chaerephon pumilus* | 7 | 2 | *Chaerephon alphaCoV/Kenya/KY22/2006* | *Eidolon bat betaCoV Kenya/KY24/2006* |
| *Eidolon helvum* | 0 | 168 |  | *Eidolon bat betaCoV Kenya/KY24/2006* |
| *Hipposideros sp.* | 0 | 3 |  | New betaCoV  New betaCoV related to SARS CoV |
|  |  |  |  |  |
| *Lissonycteris angolensis* | 0 | 1 |  | New betaCoV |
| *Mops condylurus* | 7 | 0 | *Chaerephon alphaCoV/Kenya/KY22/2006*  *Chaerephon alphaCoV/Kenya/KY41/2006*  New alphaCoV |  |
| *Pipistrellus cf. hesperidus* |  | 1 |  | A strain of Middle East Respiratory Syndrome CoV |
| *Rhinolophus cf. clivosus* | 5 | 9 | *Human alphaCoV 229E*  New alphaCoV | New betaCoV related to SARS CoV  New betaCoV |
| *Rousettus aegyptiacus* | 0 | 4 |  | Kenya bat betaCoV BtKY56/BtKY55  Bat betaCoV HKU9 |

| *Triaenops persicus* | 22 | 3 | New alphaCoV  New alphaCoV related to the NL63 CoV | *Eidolon bat betaCoV Kenya/KY24/2006* |
| --- | --- | --- | --- | --- |

* CoV = coronavirus
