# Supplementary material for: Reproduction of East-African bats may guide risk mitigation for coronavirus spillover
Source: One Health Outlook. 2020 Feb 7;2:2. doi: 10.1186/s42522-019-0008-8 (PMC7149079; doi:10.1186/s42522-019-0008-8)

**Additional file 5**

Results of the model to assess the association between CoV shedding and the “Recent weaning” season in *Eidolon helvum* and microbats.

**Figure A5.1. Posterior predictive distributions of coronavirus positive bats (histograms) and the observed coronavirus positive bats (vertical lines). The histograms show the distribution of four thousand predictions of detection in the sampled bats. The lines show the observed detection. A) Coronavirus detection across all bats. B) Coronavirus detection across age categories: adults (light blue) and non-adults (yellow). C) Coronavirus detection across the reproductive seasons: “Not recent weaning” (light blue) and “Recent weaning” (yellow). D) Coronavirus detection across the reproductive seasons in the non-adult bats: “Not recent weaning” (light blue) and “Recent weaning” (yellow). E) Coronavirus detection across the life history seasons in the adult individuals: “Not recent weaning” (light blue) and “Recent weaning” (yellow).**

**
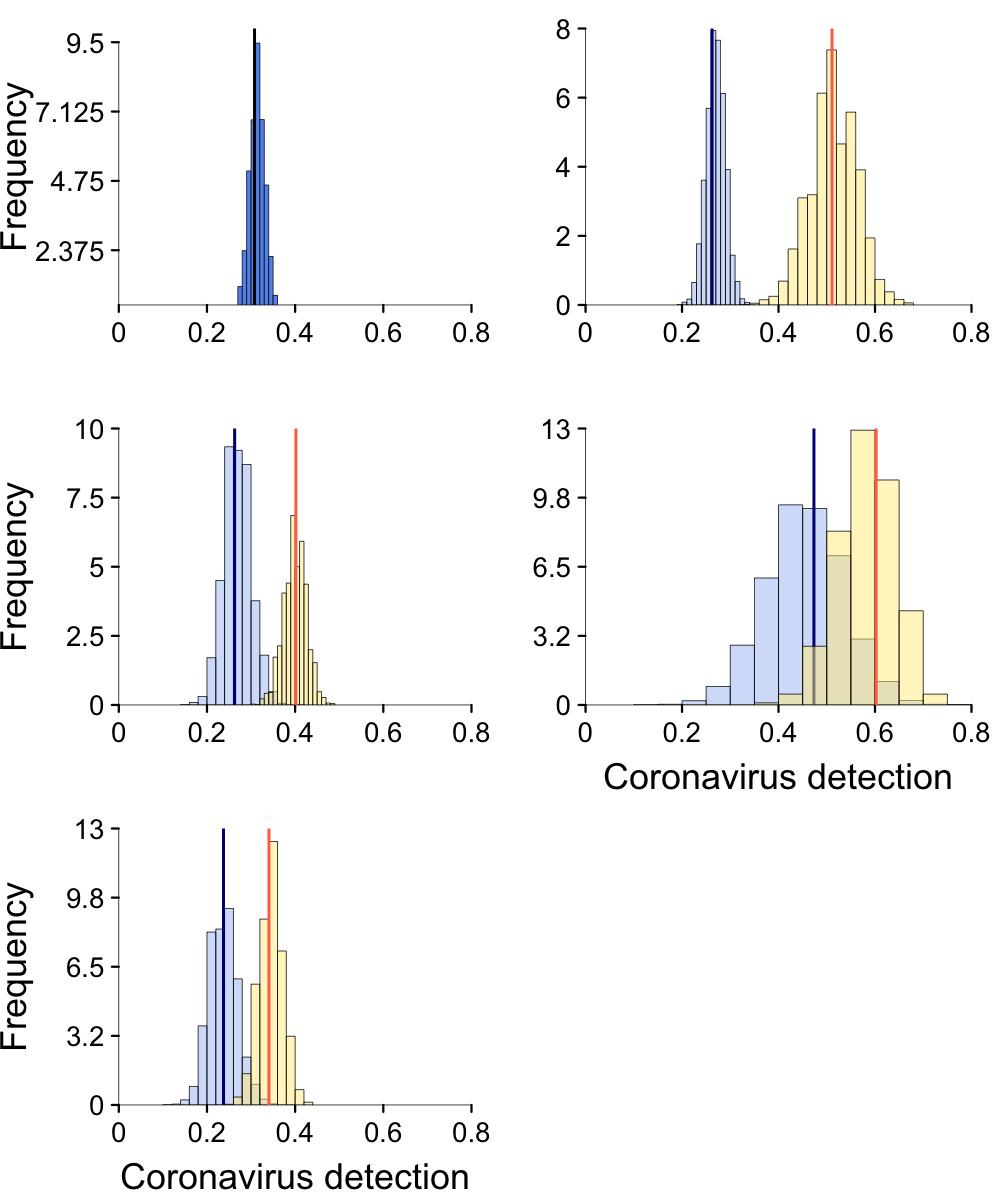
**

**Figure A5.2. The density of the coefficients’ posterior probability distributions of the selected model. RW refers to the “Recent weaning” period (versus “Not recent weaning” season).**


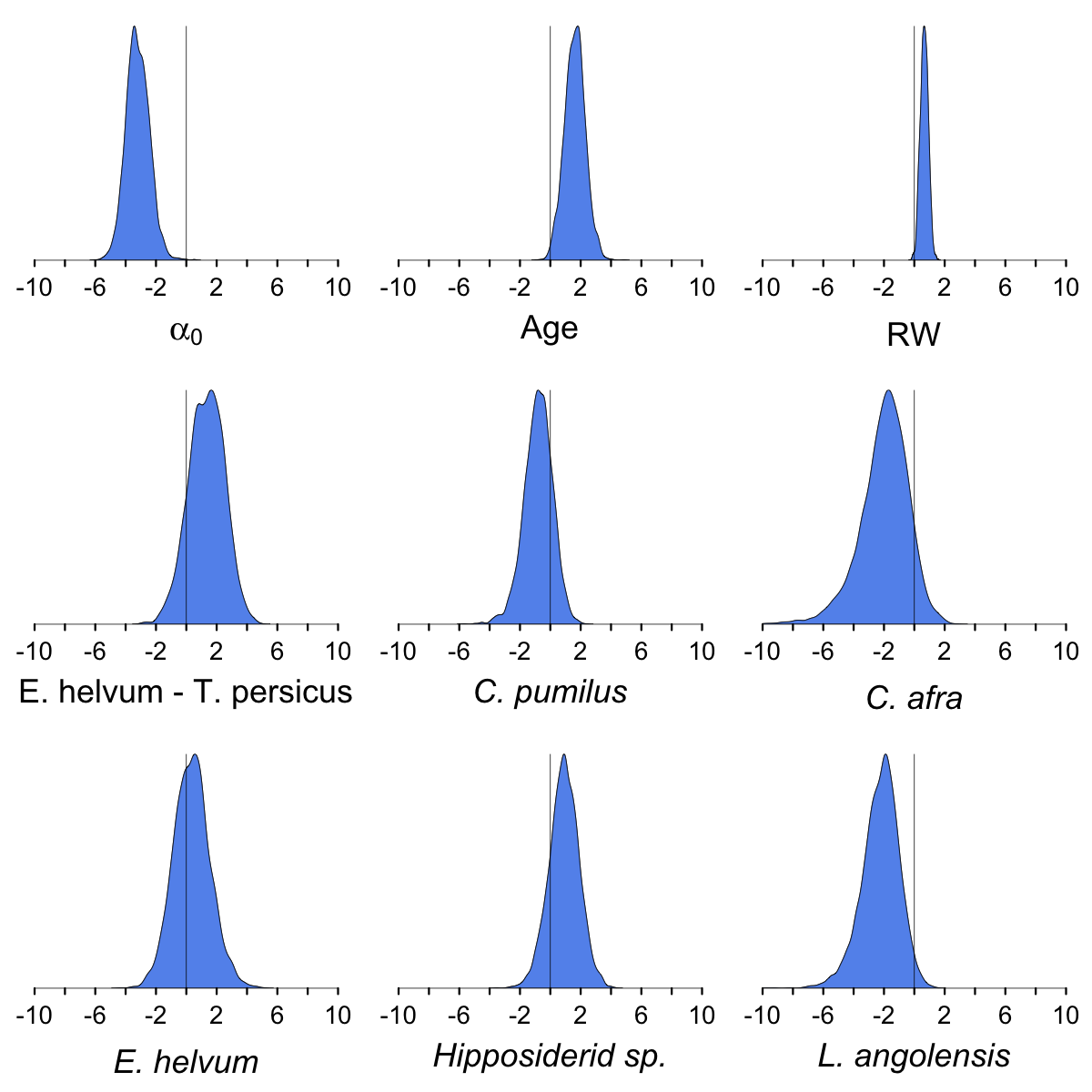


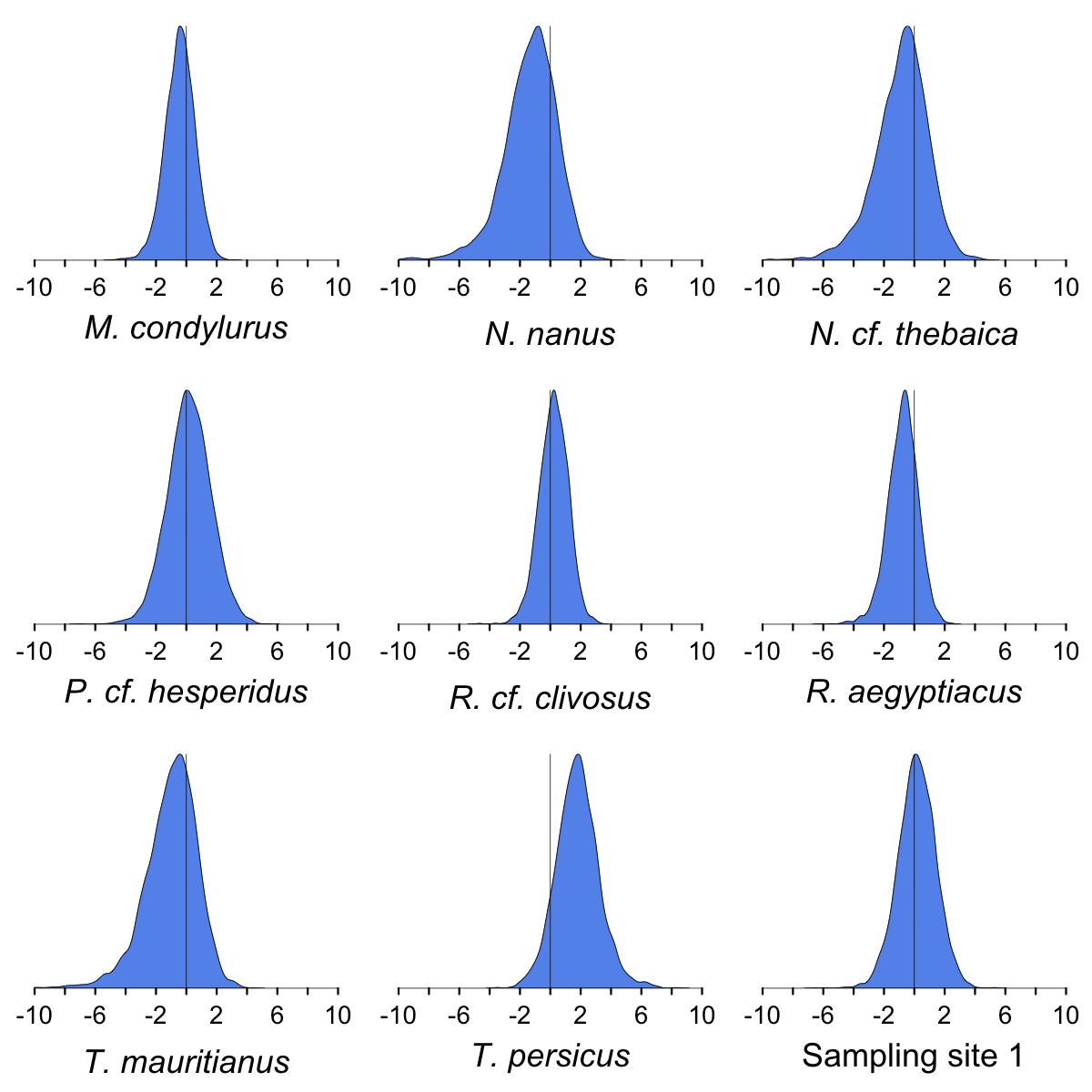


**
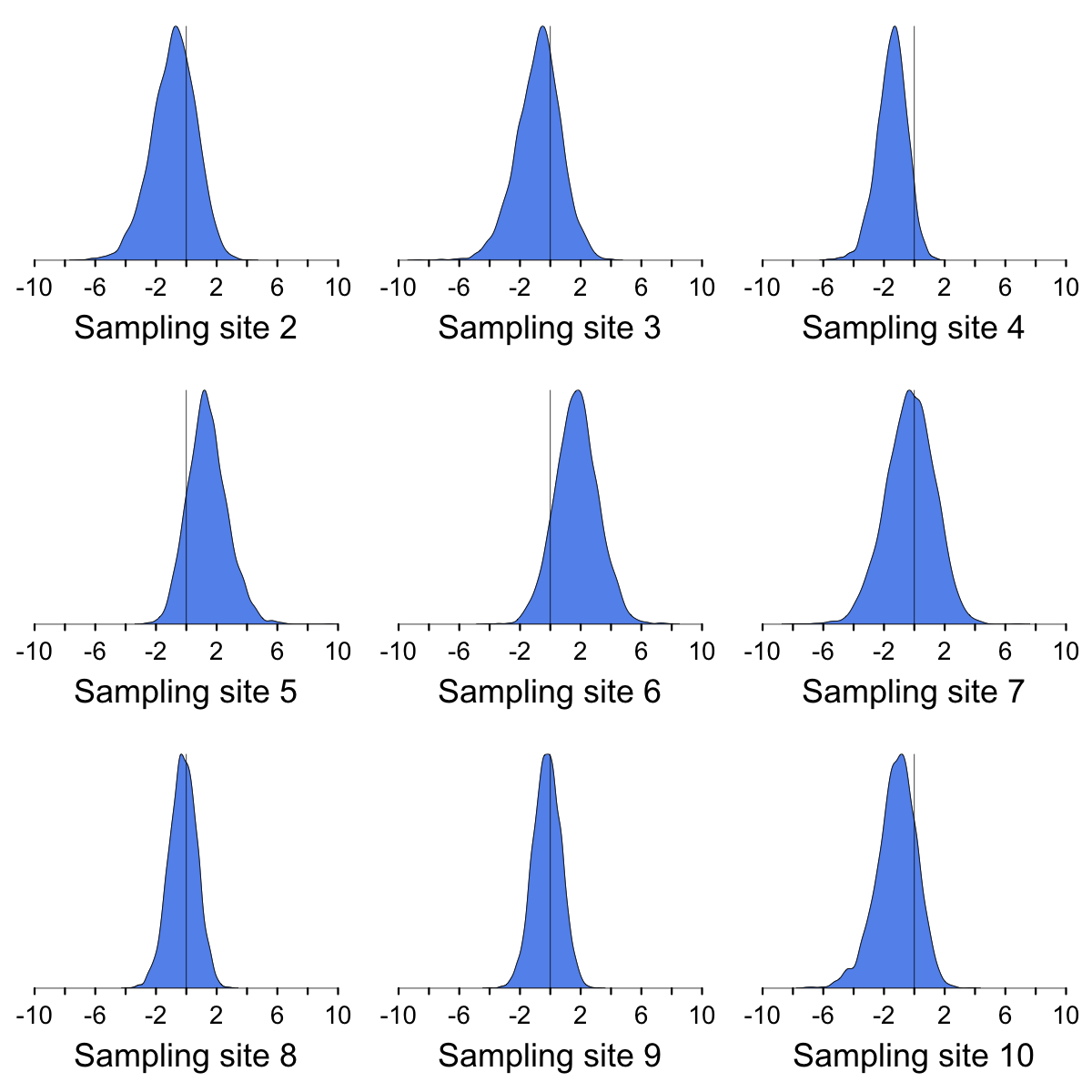
**

**
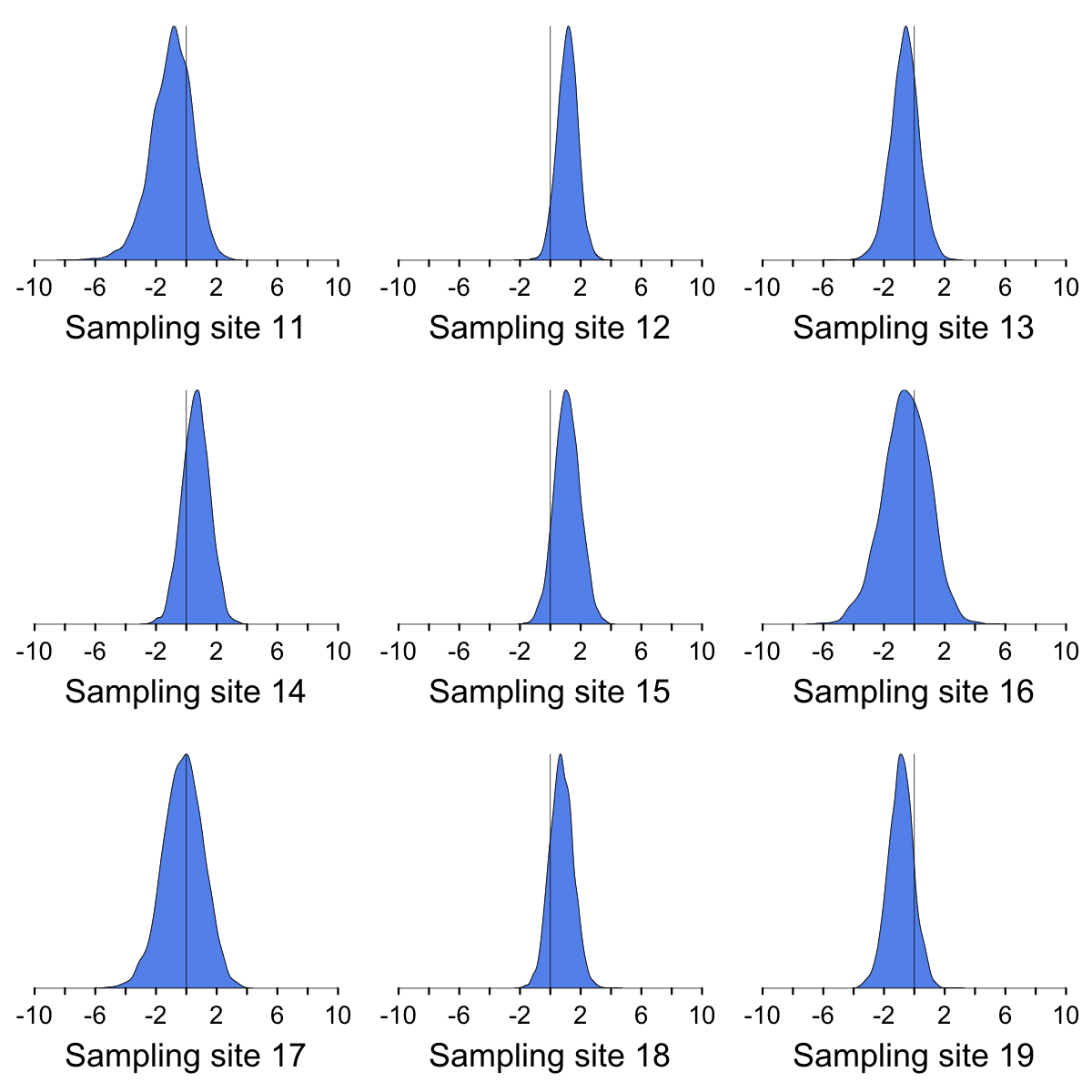
**


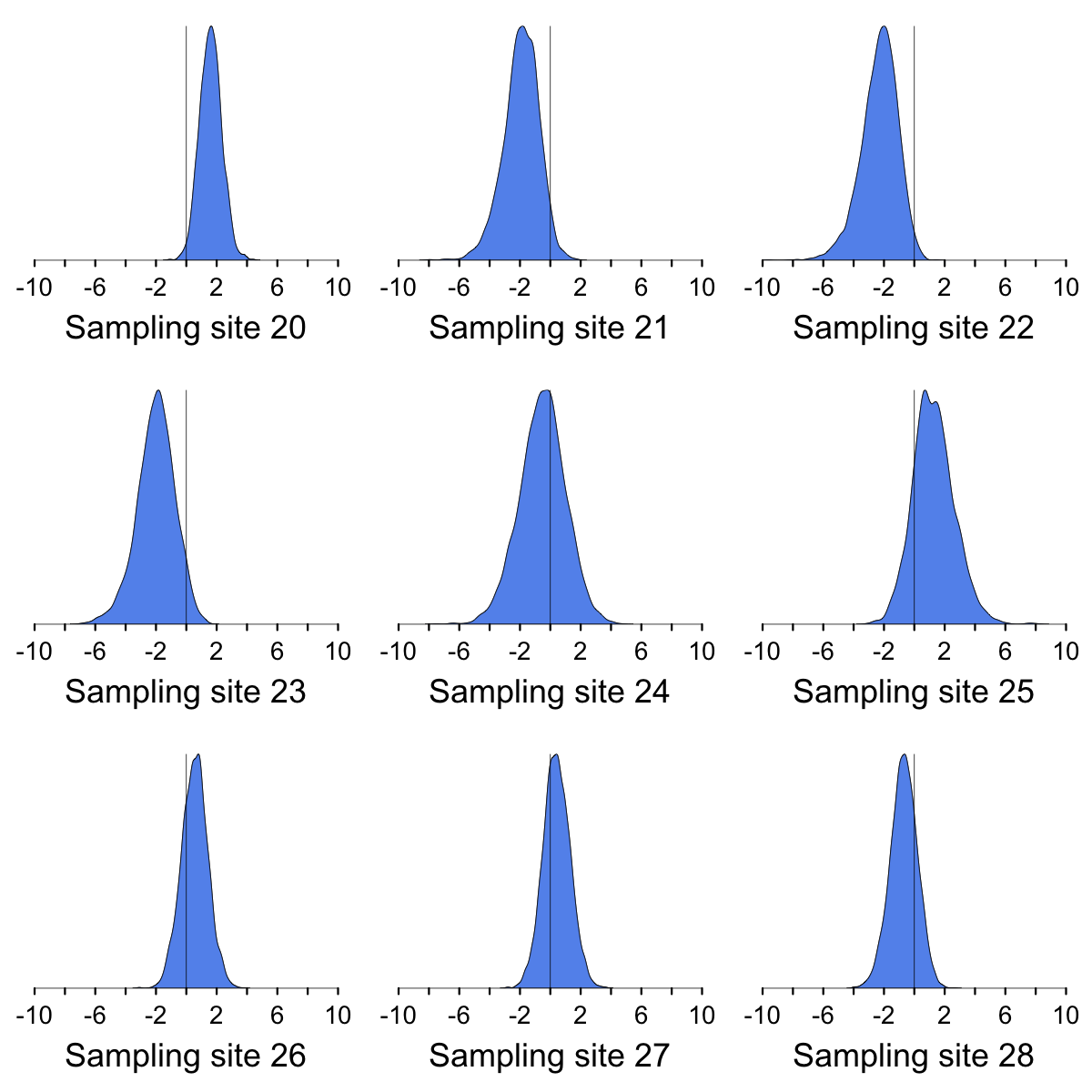


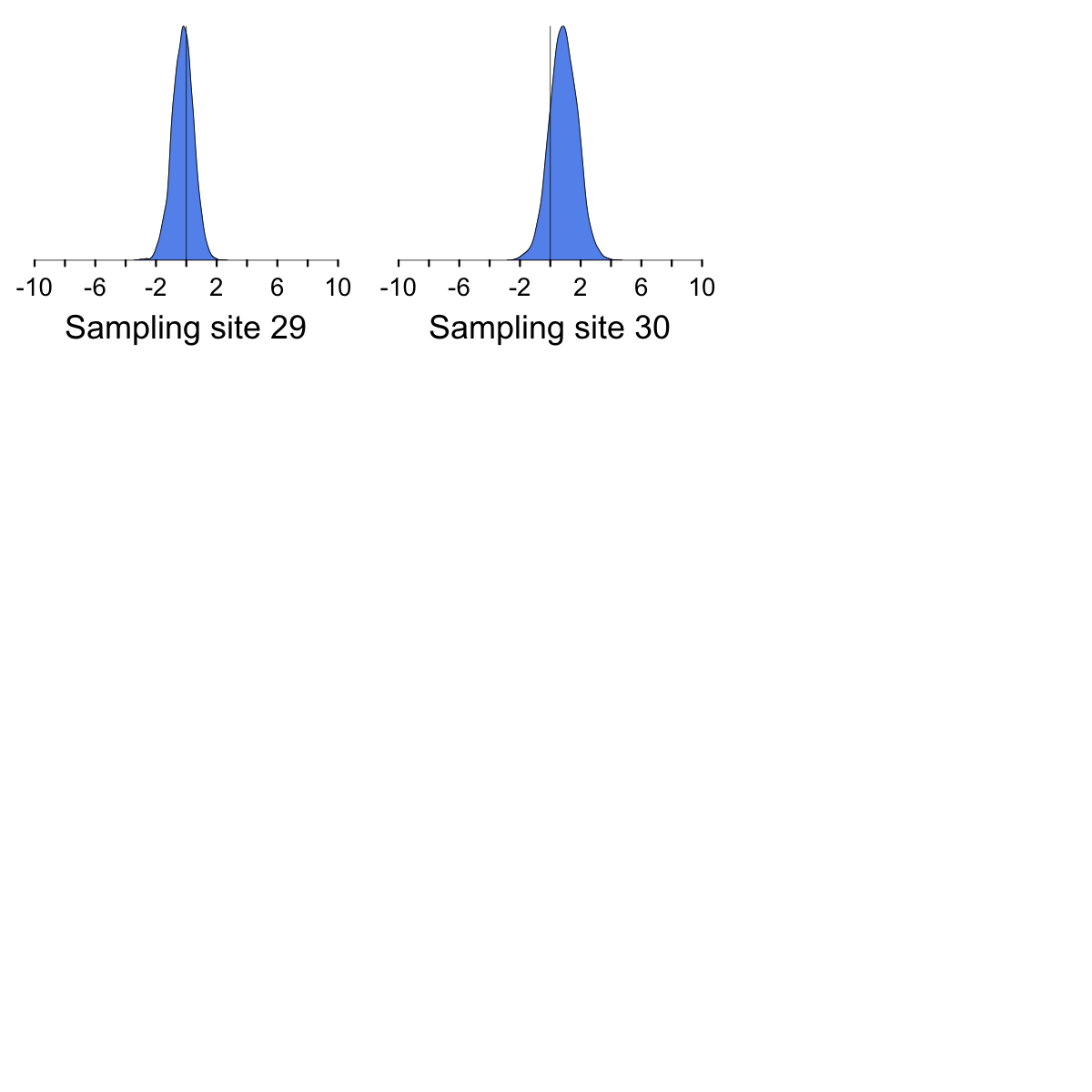

Supplement: Supplementary file 5 — Additional file 5: Results of the model to assess the association between CoV shedding and the “Recent weaning” season in Eidolon helvum and microbats. Figure S5.1. Posterior predictive distributions of coronavirus positive bats (histograms) and the observed coronavirus positive bats (vertical lines). The histograms show the distribution of four thousand predictions of detection in the sampled bats. The lines show the observed detection. A) Coronavirus detection across all bats. B) Coronavirus detection across age categories: adults (light blue) and non-adults (yellow). C) Coronavirus detection across the reproductive seasons: “Not recent weaning” (light blue) and “Recent weaning” (yellow). D) Coronavirus detection across the reproductive seasons in the non-adult bats: “Not recent weaning” (light blue) and “Recent weaning” (yellow). E) Coronavirus detection across the life history seasons in the adult individuals: “Not recent weaning” (light blue) and “Recent weaning” (yellow). Figure S5.2. The density of the coefficients’ posterior probability distributions of the selected model. RW refers to the “Recent weaning” period (versus “Not recent weaning” season). [file 42522_2019_8_MOESM5_ESM.docx]
