# Supplementary figures and images for: Reproduction of East-African bats may guide risk mitigation for coronavirus spillover
Source: One Health Outlook. 2020 Feb 7;2:2. doi: 10.1186/s42522-019-0008-8 (PMC7149079; doi:10.1186/s42522-019-0008-8)

**Additional file 6**

Sampling events -specific random intercepts values.

**
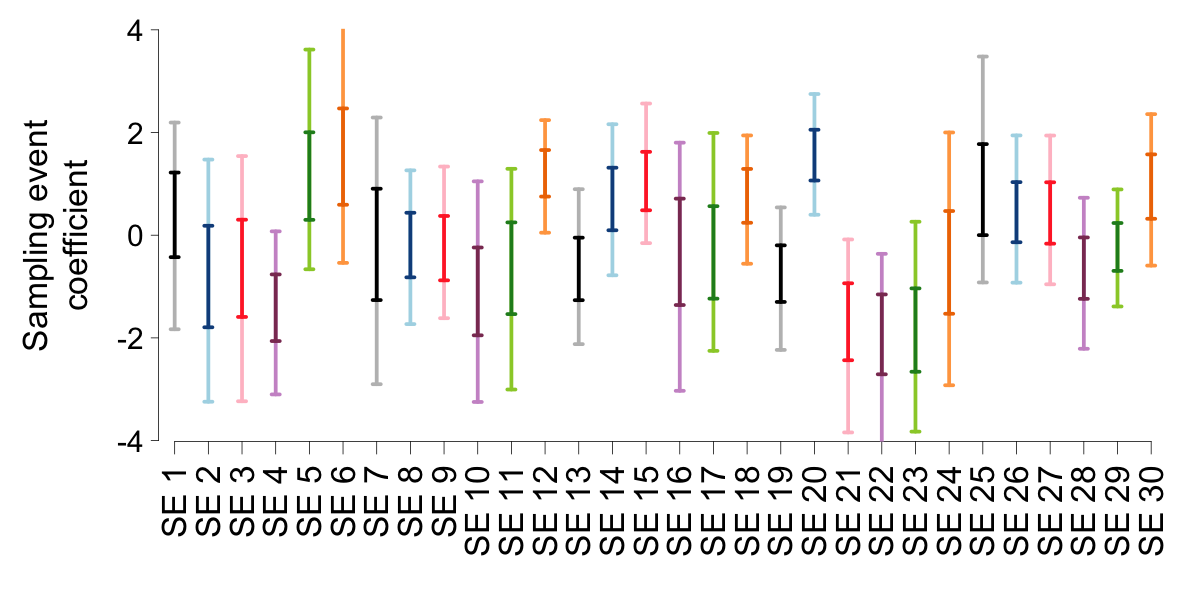
**

SE = sampling event

Supplement: Supplementary file 6 — Additional file 6: Sampling events -specific random intercepts values. [file 42522_2019_8_MOESM6_ESM.docx]
